# Improving access to mental health care: a system dynamics model of direct access to specialist care and accelerated specialist service capacity growth

**DOI:** 10.5694/mja2.51903

**Published:** 2023-03-27

**Authors:** Catherine Vacher, Adam Skinner, Jo‐An Occhipinti, Sebastian Rosenberg, Nicholas Ho, Yun Ju Christine Song, Ian B Hickie

**Affiliations:** ^1^ The University of Sydney Sydney NSW; ^2^ Computer Simulation and Advanced Research Technologies (CSART) Sydney NSW

**Keywords:** Mental health policy, Psychology, Cognitive therapy, Models, statistical, Mental health services

## Abstract

**Objective:**

To simulate the impact on population mental health indicators of allowing people to book some Medicare‐subsidised sessions with psychologists and other mental health care professionals without a referral (direct access), and of increasing the annual growth rate in specialist mental health care capacity (consultations).

**Design:**

System dynamics model, calibrated using historical time series data from the Australian Bureau of Statistics, HealthStats NSW, the Australian Institute of Health and Welfare, and the Australian Early Development Census. Parameter values that could not be derived from these sources were estimated by constrained optimisation.

**Setting:**

New South Wales, 1 September 2021 – 1 September 2028.

**Main outcome measures:**

Projected mental health‐related emergency department presentations, hospitalisations following self‐harm, and deaths by suicide, both overall and for people aged 15–24 years.

**Results:**

Direct access (for 10–50% of people requiring specialist mental health care) would lead to increases in the numbers of mental health‐related emergency department presentations (0.33–1.68% of baseline), hospitalisations with self‐harm (0.16–0.77%), and deaths by suicide (0.19–0.90%), as waiting times for consultations would increase, leading to disengagement and consequently to increases in adverse outcomes. Increasing the annual rate of growth of mental health service capacity (two‐ to fivefold) would reduce the frequency of all three outcomes; combining direct access to a proportion of services with increased growth in capacity achieved substantially greater gains than an increase in service capacity alone. A fivefold increase in the annual service growth rate would increase capacity by 71.6% by the end of 2028, compared with current projections; combined with direct access to 50% of mental health consultations, 26 616 emergency department presentations (3.6%), 1199 hospitalisations following self‐harm (1.9%), and 158 deaths by suicide (2.1%) could be averted.

**Conclusion:**

The optimal combination of increased service capacity growth (fivefold) and direct access (50% of consultations) would have double the impact over seven years of accelerated capacity growth alone. Our model highlights the risks of implementing individual reforms without knowledge of their overall system effect.

**The known:** About 53% of people with mental disorders in Australia do not receive professional mental health care. Medicare‐subsidised access to services delivered by psychologists and mental health allied professionals requires assessment and referral by a medical practitioner.

**The new:** Allowing people to book subsidised care without a referral would lead to poorer population mental health outcomes because of longer waiting times. A combination of direct access with accelerated service capacity growth would achieve significantly greater benefits than increasing capacity alone.

**The implications:** Providing direct access to Medicare‐subsidised mental health care would improve population mental health indicators only when combined with large increases in specialist mental health care service capacity.

Mental disorders are among the top ten causes of illness burden worldwide.^1^ However, more than half the people in Australia who have mental disorders do not receive professional mental health care,^2^ causing considerable avoidable distress and illness for those directly affected and their families. A meta‐analysis of 37 surveys from around the world found that the median treatment gap for schizophrenia comprises 33%, for depression 56%, for anxiety 57%, and for alcohol abuse and dependence 78% of people with these disorders.^3^ While there are many barriers to mental health care, particularly to specialised treatments that deliver better outcomes, accessibility, affordability, and local availability of services are critical to equitable, quality care.

In 2006–07, 65.1% of people in Australia with mental disorders did not receive professional treatment.^4^ In 2006, the Better Access initiative,^5^ based on the earlier Better Outcomes Program,^6^ provided people access to government‐subsidised sessions with psychologists and other allied professionals. The Better Access pathway requires referring doctors (typically general practitioners) to diagnose a mental disorder, prepare a mental health plan, and select an appropriate number of care sessions with psychologists or allied professionals; the patient is then entitled to a Medicare rebate for these specialist services.^5^ This policy initiative was estimated to have reduced the treatment gap to 54% by 2009–10.^7^ However, the gap has not closed further during the past decade, and was estimated to be 52.9% in 2020–21.^2^ In 2020–21, only 29.2% of people receiving care under Better Access were new clients.^8^

As reported rates of psychological distress increased during the coronavirus disease 2019 (COVID‐19) pandemic,^9^ the maximum number of Medicare‐subsidised sessions per person was temporarily raised from ten to twenty per year.^10^ However, few people were likely to reach the increased maximum number of subsidised sessions; in 2019, the mean number of subsidised sessions per users was 4.5, well below the ceiling of ten.^11^

The Australian Association of Psychologists incorporated^12^ and the Australian Psychological Society^13^ have recently advocated allowing access to subsidised care by specialist psychologists and allied professionals, such as social workers and occupational therapists, without requiring referral by a general practitioner and a mental health plan. Allowing people direct access to subsidised mental health services may remove some barriers to care, but the overall impact on mental health outcomes, and potential unintended consequences, have not been explored.

How people engage with mental health services depends on their age and level of psychological distress. They may receive treatment that reduces their psychological distress, or disengage from care because of excessive waiting time or dissatisfaction with treatment. We used dynamic systems modelling and simulation to test whether direct access to specialised mental health services would have the intended effects on indicators of population mental health, such as numbers of mental health‐related emergency department (ED) presentations, hospitalisations related to self‐harm, and deaths by suicide.

## Methods

Our system dynamics model was developed in Stella Architect 2.0 (isee Systems). The model includes causal relationships between demographic characteristics, post‐secondary education, employment, psychological distress, suicidal behaviour, and mental health services for the NSW population, and takes into account the impact of the COVID‐19 pandemic on these factors (Supporting Information, model structure; figures 1–10).

### Data sources and simulations

The model was calibrated using historical time series data from the Australian Bureau of Statistics (https://www.abs.gov.au), HealthStats NSW (https://www.healthstats.nsw.gov.au), the Australian Institute of Health and Welfare (AIHW),^14^, ^15^ and the Australian Early Development Census (https://www.aedc.gov.au/data‐explorer). Parameter values that could not be derived from these sources were estimated by constrained optimisation, using Powell's method,^16^ which minimises the mean absolute proportional deviation of model outputs from historical data (Supporting Information, table 1, figure 11).

Our model forecasts the impact on mental health indicators of enabling a proportion of subsidised specialised mental health services to be directly available without referral from a doctor (“direct access”). We also examined the impact of combining direct access with an increase in the annual growth in mental health service capacity, and the consequences of an increase in demand for mental health services if a substantial proportion of services were available by direct access, as well as combinations of these factors (Box 1).

Box 1 The mental health service provision factors simulated in our system dynamics model (default duration for each: from 1 January 2022 until end of simulation)*

**Table:** 

Factor	Description
Direct access	Enables a proportion (10–50%) of people seeking mental health care to receive Medicare‐subsidised sessions with psychologists or allied professionals without a general practitioner referral and mental health plan.
Accelerated growth in specialised mental health service capacity	Annual growth in number of sessions with specialised mental health services (psychologists, psychiatrists, mental health allied professionals) increased (by factor of two to five).
Increased demand for mental health services	Increased proportion of people who perceive a need for mental health care (5% or 10%) and seek it from a general practitioner or online services, or from psychologists and allied professionals if direct access is available.

* Further details: Supporting Information, table 2.

The impact of an intervention was defined as the difference between the baseline and intervention scenarios in the projected cumulative number of events for the period 1 September 2021 – 1 September 2028. The intervention scenarios were modelled separately for the general New South Wales population (all ages) and for people aged 15–24 years. The model was initially run without taking into account the impact of COVID‐19 on population mental health; all subsequent runs included its impact, including temporary social dislocation and altered labour transitions (Supporting Information, table 3). The baseline scenario included two temporary Australian government responses to COVID‐19: an economic initiative to limit unemployment (the JobKeeper wage subsidy program^17^) and the increase in the annual maximum number of Medicare‐subsidised psychologist and allied professional care sessions from ten to twenty (extended Better Access program; ended 31 December 2022^10^). Population mental health outcomes were projected as numbers of mental health‐related ED presentations, hospitalisations following self‐harm, and deaths by suicide, as well as the prevalence of moderate to very high psychological distress, assessed with the Kessler Psychological Distress Scale (K10);^18^ scores of 16–50 were deemed to indicate moderate to very high psychological distress.

### Ethics approval

As our simulations were based on publicly available data, we did not seek ethics approval for our study.

## Results

### Direct access alone

According to our model, the greatest impact of allowing direct access to a proportion of people using Medicare‐subsidised specialised mental health services on population mental health indicators would be on mental health‐related ED presentations: across the 7‐year period projected, the number would increase by 2464 presentations (0.33% of baseline number) if 10% of people seeking mental health care had direct access, and by 12 439 presentations (1.68%) with direct access to 50% of consultations. The numbers of hospitalisations with self‐harm and deaths by suicide would also increase (Box 2). According to our model, these unexpected effects were attributable to direct access causing longer waiting times for appointments with psychologists and other specialist providers, leading to disengagement, psychological distress, and poorer mental health outcomes. We therefore investigated combining direct access with an increase in specialist service capacity growth.

Box 2 Simulated impact of allowing direct access to a proportion of Medicare‐subsidised specialised mental health services on population mental health indicators, 1 September 2021 – 1 September 2028*

**Table:** 

		Proportion of direct access consultations		
Outcome	Baseline number	10%	20%	50%
Emergency department presentations (mental health): all ages	738 913	+2464 (0.33%)	+5192 (0.7%)	+12 439 (1.68%)
Emergency department presentations (mental health): 15–24 years	141 806	+97 (0.07%)	+230 (0.16%)	+645 (0.45%)
Hospitalisations with self‐harm: all ages	63 198	+101 (0.16%)	+207 (0.33%)	+487 (0.77%)
Hospitalisations with self‐harm: 15–24 years	18 453	+4 (0.02%)	+10 (0.06%)	+30 (0.16%)
Deaths by suicide: all ages	7433	+14 (0.19%)	+28 (0.38%)	+67 (0.90%)
Deaths by suicide: 15–24 years	886	+0.2 (0.02%)	+0.5 (0.06%)	+1.5 (0.16%)

* Assumptions: extended Better Access (maximum twenty sessions per year) available until 31 December 2022; direct access starts 1 January 2022.

### Accelerated growth of specialist mental health care capacity

We simulated increasing the annual rate of growth in the number of available specialised mental health care consultations (with psychologists, psychiatrists, and mental health allied workers) by a factor of two, three, or five. The baseline annual capacity growth in specialist consultations was modelled as a yearly linear increase calibrated using AIHW data on Medicare‐subsidised mental health‐specific services;^15^ during 2021–2028, the baseline growth would be similar to a mean 3% annual increase in capacity. Doubling the annual growth in consultations was projected to prevent 7083 mental health‐related ED presentations (0.96% of baseline number), 326 hospitalisations with self‐harm (0.52%), and 43 deaths by suicide (0.58%) across seven years; increasing it fivefold would prevent 12 806 ED presentations (1.73%), 562 hospitalisations (0.89%), and 78 deaths by suicide (1.1%) (Box 3), and was forecast to reduce the prevalence of moderate to very high psychological distress in 2028 from 40.24% to 39.48% (Box 4).

Box 3 Simulated impact of direct access to a proportion of Medicare‐subsidised specialised mental health services, together with more rapid growth of specialised service capacity, on population mental health indicators, 1 September 2021 – 1 September 2028*

**Table:** 

	Scenario							
**Mental health services growth**	**× 2**	**× 2**	**× 3**	**× 3**	**× 3**	**× 5**	**× 5**	**× 5**
**Direct access proportion**	**—**	**10%**	**—**	**20%**	**20%**	**—**	**50%**	**50%**
**Perceived need for mental health services**	**—**	**—**	**—**	**—**	**+5%**	**—**	**—**	**+10%**
ED presentations (mental health): all ages	–7083 (0.96%)	–8469 (1.15%)	–10 324 (1.40%)	–16 355 (2.21%)	–13 485 (1.83%)	–12 806 (1.73%)	–26 616 (3.60%)	–22 439 (3.04%)
ED presentations (mental health): 15–24 years	–825 (0.58%)	–1120 (0.79%)	–1082 (0.76%)	–2064 (1.46%)	–1602 (1.13%)	–1268 (0.89%)	–3136 (2.21%)	–2446 (1.72%)
Hospitalisations with self‐harm: all ages	–326 (0.52%)	–368 (0.58%)	–469 (0.74%)	–721 (1.14%)	–732 (1.16%)	–562 (0.89%)	–1199 (1.90%)	–1295 (2.05%)
Hospitalisations with self‐harm: 15–24 years	–47 (0.26%)	–61 (0.33%)	–58 (0.31%)	–116 (0.63%)	–127 (0.69%)	–64 (0.35%)	–176 (0.96%)	–213 (1.15%)
Deaths by suicide: all ages	–43 (0.58%)	–48 (0.64%)	–64 (0.86%)	–94 (1.3%)	–95 (1.3%)	–78 (1.1%)	–158 (2.13%)	–170 (2.28%)
Deaths by suicide: 15–24 years	–2.3 (0.26%)	–2.9 (0.33%)	–2.8 (0.31%)	–5.6 (0.63%)	–6.1 (0.69%)	–3 (0.35%)	–9 (1.0%)	–10 (1.2%)

ED = emergency department.

* Assumptions: extended Better Access (maximum twenty sessions per year) available until 31 December 2022; more rapid growth in specialised mental health services commences 1 January 2022; direct access and increase in perceived needs for mental health services commence 1 January 2024.

Box 4 Simulated impact of direct access to a proportion of Medicare‐subsidised specialised mental health services, together with more rapid growth of specialised service capacity, on the prevalence of moderate to very high psychological distress in the general population in September 2028*

**Table:** 

	Scenario (combination of interventions)							
**Mental health services growth**	**× 2**	**× 2**	**× 3**	**× 3**	**× 3**	**× 5**	**× 5**	**× 5**
**Direct access proportion**	**—**	**10%**	**—**	**20%**	**20%**	**—**	**50%**	**50%**
**Perceived need for services**	**—**	**—**	**—**	**—**	**+5%**	**—**	**—**	**+10%**
**Prevalence of moderate to very high psychological distress** ^†^								
All people								
Prevalence (baseline: 40.24%)^‡^	39.52%	39.34%	39.48%	38.54%	38.45%	39.48%	37.95%	37.50%
Difference (percentage points)	–0.72	–0.90	–0.76	–1.70	–1.79	–0.76	–2.30	–2.74
People aged 15–24 years								
Prevalence (baseline: 52.87%)^‡^	52.45%	52.17%	52.48%	51.67%	51.45%	52.46%	51.39%	50.86%
Difference (percentage points)	–0.42	–0.70	–0.39	–1.20	–1.42	–0.41	–1.48	–2.01

* Assumptions: extended Better Access (maximum twenty sessions per year) available until 31 December 2022; more rapid growth in specialised mental health services commences 1 January 2022; direct access and increase in perceived needs for mental health services commence 1 January 2024.

† Kessler Psychological Distress Scale (K10) score of 16–50.

‡ The prevalence of moderate to very high psychological distress in New South Wales increased from 2013 (all ages, 27.2%; 16–24 years, 33.5%) to 2019 (all ages, 43.5%; 16–24 years, 63.5%).^19^

### Combining direct access with accelerated capacity growth

We then combined more rapid capacity growth with different degrees of direct access to mental health care (start of direct access moved to 1 January 2024 to allow growth in service capacity). The numbers of adverse outcomes averted were larger than for more rapid service growth alone. If direct access to 50% of consultations for people seeking help was combined with a fivefold increase in service capacity growth, the proportions of mental health‐related ED presentations averted during 2021–2028 increased from 1.73% (increased capacity alone) to 3.60%, of averted hospitalisations with self‐harm from 0.89% to 1.90%, and of averted deaths by suicide from 1.1% to 2.1% (Box 3). The prevalence of moderate to very high psychological distress in 2028 decreased from 39.48% to 37.95% (Box 4). The improvements were even more marked for people aged 15–24 years, but the proportions of averted events were smaller than the overall figures for all ages (Box 3). The proportions of averted adverse outcomes were slightly larger if the extended Better Access program (ten additional sessions per year) were retained until the end of December 2023, as the extended program benefited from the increased service capacity (Supporting Information, table 4).

For the combination of 50% direct access and fivefold increase in service capacity growth, a 10% increase in demand for mental health services reduced the proportion of mental health‐related ED presentations from 3.60% (without increased demand) to 3.04%, and slightly increased the proportions of averted hospitalisations with self‐harm (from 1.90% to 2.05%) and of averted deaths by suicide (from 2.13% to 2.28%) (Box 3).

With a doubling of the annual rate of specialist service capacity increase, the optimal direct access proportion for averting mental health‐related ED presentations in our model was 10% (1.15% of admissions averted), and beyond 20% the benefit was less than that achieved by doubling capacity growth alone. With a tripling of the services growth rate, the optimal direct access proportion was 20% (2.21% of admissions averted); with a fivefold increase in the growth rate, the estimated optimal direct access proportion was 50% (3.60% of admissions averted) (Box 5).

Box 5 Simulated impact of direct access to a proportion of Medicare‐subsidised specialised mental health services and more rapid growth of specialised service capacity on the proportion of averted mental health‐related emergency department presentations, 1 September 2021 – 1 September 2028*

**Figure:**
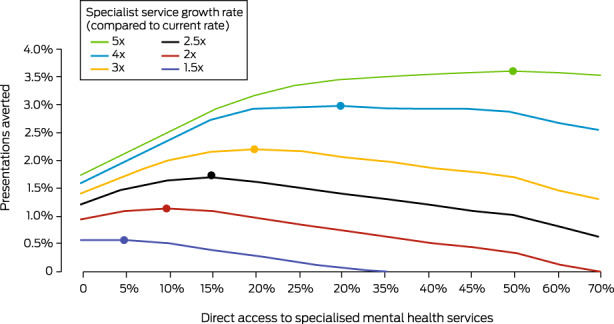
* The optimal combination of direct access and accelerated services growth for averting emergency department admissions is marked by a solid circle on each services growth curve; beyond this point, services system capacity is saturated.

## Discussion

We used dynamic systems modelling to simulate the impact of allowing people seeking mental health care to directly book Medicare‐subsidised consultations with psychologists and other mental health care professionals. Our key findings were that direct access must be paired with an increase in specialist service capacity, and that the optimal direct access proportion depends on the level of accelerated specialist service capacity growth. The model projected that even limited direct access (as few as 10% of consultations) would lead to larger numbers of mental health‐related ED presentations, hospitalisations with self‐harm, and deaths by suicide over a seven‐year period. This unexpected result was explained by increased waiting times for specialised care, leading to disengagement, greater psychological distress, and poorer mental health outcomes.

In contrast, combining limited direct access with substantially increased specialised mental health service growth was projected to avert adverse outcomes to a greater degree than increasing service capacity alone. For example: with a fivefold increase in the annual service growth rate, which would increase capacity by 71.6% (compared with the baseline scenario) by the end of the 7‐year period modelled, and allowing 50% of people to have direct access to mental health consultations, the proportions of adverse outcomes averted were each twice as large as with fivefold growth in service capacity alone.

In an earlier dynamic model analysis, we noted that the mental health specialist service capacity in Australia is inadequate for meeting demand, particularly specialist psychologist services.^20^ Receiving the right care when it is first sought enhances the prospects for recovery, while disengagement from a saturated mental health care system leads to distress and deteriorating mental health.^21^ Without expanding the capacity of specialist psychology services, removing the requirement for consultations with a general practitioner to obtain a referral exposes people seeking help to the risk of longer waiting times. While direct access to psychologists is unlikely to lead to markedly increased demand for specialised mental health services, given increasing out‐of‐pocket costs for such care,^22^ our modelling suggests that optimal combinations of direct access and increased service capacity growth could achieve better results than increased service capacity alone, even were demand for services to increase moderately. On the other hand, the benefits of direct access cannot be realised without remediating service capacity constraints.

Many potential benefits and drawbacks to direct access to psychologists and other specialist practitioners were not considered in our modelling study. General and other medical practitioners, particularly paediatricians, psychiatrists, and neurologists, can diagnose and treat conditions that can underlie mental problems, including neurological disorders, nutrient deficiencies, endocrine disorders, and auto‐immune conditions. General practitioners can also prescribe psychotropic medications, and they provide the best path to therapy for people who also have physical health problems by coordinating their health care needs. On the other hand, direct access could facilitate earlier access to specialised treatment for people unable to discuss their problems in a brief, medically focused general practice consultation, are discouraged because they perceive the gatekeeper role of general practitioners as an administrative hurdle, worry that revealing mental health problems could lead to their physical health problems being dismissed as psychosomatic,^23^ or fear stigmatisation following a formal mental health diagnosis or the preparation of a mental health plan.

The 2020 Productivity Commission inquiry into mental health care found that the structuring of Medicare subsidies for mental health services could be more effective than the current “one size fits all” model.^11^ Increasing access to mental health care is important, but providing data on the effectiveness of care and directing services appropriately are crucial. Reforms of the Better Access program, which was not designed for people with persistent or more complex disorders, are being considered by the Australian government.^24^ The needs of people with more complex problems may be better met by increasing their access to effective, specialist multidisciplinary care, which may include social and vocational support.^25^ Using technology to support mental health care can improve the coordination of patient care and increase the efficiency of the available mental health service capacity.

### Limitations

Most inputs for our model were derived from population health survey data, published demographic and economic data, and Medicare Benefits Schedule data that may vary in quality. Values for parameters not available from such sources were estimated by constrained optimisation and local verification.

### Conclusions

Our study illustrates the capacity of dynamic systems modelling to support policy planning, as well as the risks attached to implementing single policy elements without understanding their implications when introduced into a complex system. Economic analyses of the modelled policies are still required. Providing direct access to Medicare‐subsidised mental health care improves population mental health outcomes only when combined with large increases in specialist mental health care service capacity.

## Open access

Open access publishing facilitated by The University of Sydney, as part of the Wiley ‐ The University of Sydney agreement via the Council of Australian University Librarians.

## Competing interests

Jo‐An Occhipinti is head of Systems Modelling, Simulation and Data Science at the Brain and Mind Centre (University of Sydney) and managing director of Computer Simulation and Advanced Research Technologies (CSART). Ian Hickie is the Co‐Director (Health and Policy) at the Brain and Mind Centre (BMC). The BMC provides early intervention youth services under contract with headspace. Ian Hickie is the Chief Scientific Advisor to and a 3.2% equity shareholder in InnoWell Pty Ltd. InnoWell was formed by the University of Sydney (45% equity) and PwC (Australia; 45% equity) to deliver the $30 million Australian government‐funded Project Synergy (2017–20) for the transformation of mental health services, and to lead transformation of mental health services internationally through the use of innovative technologies.

## Data sharing

Please contact Catherine Vacher with requests to access the model file.

## Supporting information


Supplementary methods.
