# Supplementary material for: Improving access to mental health care: a system dynamics model of direct access to specialist care and accelerated specialist service capacity growth
Source: Med J Aust. 2023 Mar 27;218(7):309–14. doi: 10.5694/mja2.51903 (PMC10952178; doi:10.5694/mja2.51903)
Supplement: Supplementary file 1 — Supplementary methods. [file MJA2-218-309-s001.pdf]

The Medical Journal of Australia

Supporting Information

Supplementary methods

**This appendix was part of the submitted manuscript and has been peer reviewed.
It is posted as supplied by the authors.**

Appendix to: Vacher C, Skinner A, Occhipinti JA, et al. Improving access to mental health care: a system dynamics model of direct access to specialist care and accelerated specialist service capacity growth. *Med J Aust* 2023; doi: 10.5694/mja2.51903.

Model structure

a) Overview

The system dynamics model was developed using Stella Architect 2.0. The model captures the causal relationships between population, demographics, post-secondary education, employment, psychological distress, suicidal behaviour and mental health services in NSW and incorporates the impact of the coronavirus disease 2019 (COVID-19) pandemic on these components. Figure 1 shows a high-level overview of the causal structure and pathways of the system dynamics model.

The system dynamics model used for study consists of interconnected components, or sectors, that includes:

1- A population sector, which captures changes in population size and structure as a consequence of births, migration, aging, and mortality;

2- A psychological distress sector that models the onset of and recovery from moderate to very high psychological distress as measured by the Kessler Psychological Distress Scale (K10)[1];

3- A developmental vulnerability sector modelling the increased risk of developmentally vulnerable children developing mental health disorders as they grow into adulthood;

4- A suicidal behaviour sector that captures numbers of self-harm hospitalisations and suicides;

5- An education sector that captures the commencement, discontinuation, and completion of post-secondary education and vocational studies;

6- An employment sector, that models the flows between employment, underemployment, unemployment, and not participating in the labour force;

7- A mental health services sector, that captures the flow of psychologically distressed, help-seeking people through various services pathways, including general practitioners, psychologists, allied health professionals, psychiatrists, emergency departments, general and psychiatric hospitals, and community mental health services.

The impact of COVID-19 is modelled as a temporary effect of the pandemic and associated public health orders on labour transitions, social dislocation, access to services and rates of psychological distress.

Note that given the large number of variables in the model, the model description herein is an overview that does not exhaustively cover all stocks, flows and variables. The level of detail provided here is, however, sufficient to allow interested readers to critically assess the internal structure of all core model components and their interconnections.

Figure 1. Overview of the causal structure of the system dynamics model

The bolded arrows represent the main causal pathways involved in the tested scenarios. The plus and minus signs indicate the effect. COVID-19 temporarily decreases migration, employment, and access to services, while increasing psychological distress. Developmental vulnerability and unemployment increase psychological distress, which increases demand for mental health services. Mental health services decrease suicidal behaviour and psychological distress, except if people disengage from services, in which case the psychological distress increases. Psychological distress increases suicidal behaviour, which in turn increase demand for services.

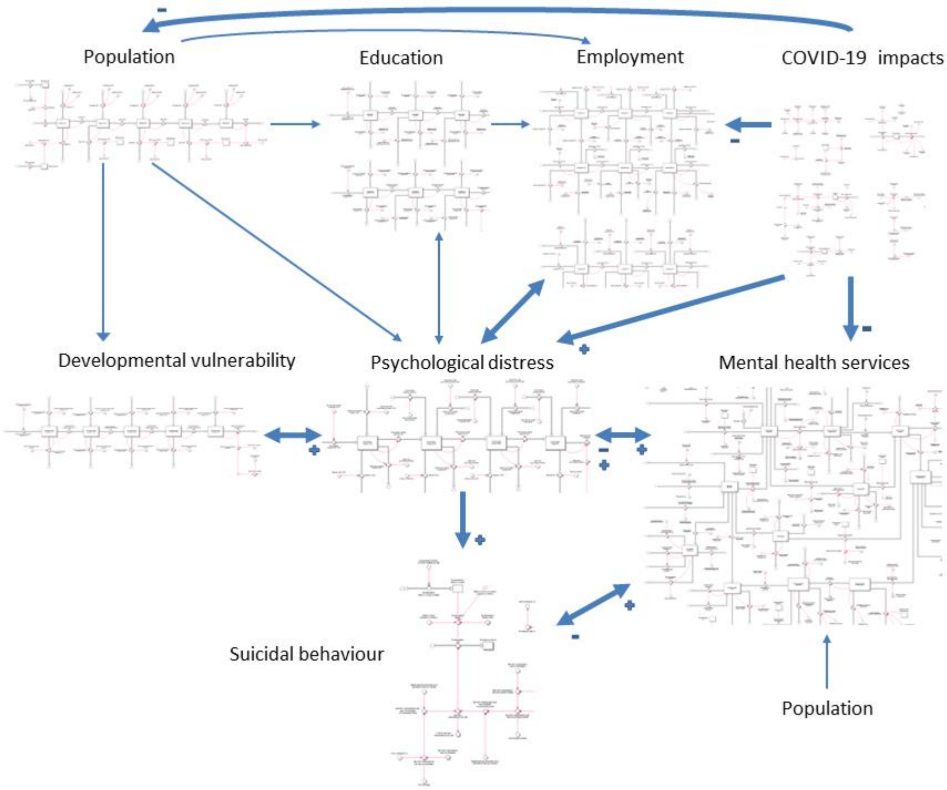

The model was calibrated using historical, time-series data from the Australian Bureau of Statistics (population sizes, birth and mortality rates, overseas and internal migration rates (1), employment rates and transitions (2, 3), education and non-school qualification data (4), family characteristics (5), and the impact of the pandemic on psychological distress prevalence (6)), HealthStats NSW (psychological distress prevalence (7), intentional self-harm hospitalizations (8) and suicide deaths (9)), the Australian Institute of Health and Welfare (AIHW) (mental-health-related ED presentations (10), and mental health service usage statistics (11)), and the Australian Early Development Census (childhood developmental vulnerability prevalence data (12)). Parameters that could not be derived from available data were estimated by constrained optimisation.

b) Population sector

The population sector (figure 2) models the changes in population sizes across different age groups as a result of births, migration, aging, and mortality. The total national population is represented as 5 stocks (state variables), corresponding to numbers of people aged 0−14 years, 15−24 years, 25−44 years, 45−64 years, and 65 years and above. The size of the population increases via births (which flow into the stock of 0−14-year-olds) and immigration and decreases through emigration and mortality. Aging is modelled as a first-order delay, in which people flow out of each stock (except the stock of people aged ≥ 65 years) at a rate n/d, where n is the number of people in the stock at any particular time point and the delay time d is the mean number of years a person spends in the stock. Births occur at the rate bP, where b is the per capita birth rate and P the total population. Deaths occur at the rate M_iP_i, where M_i and P_i are respectively the mortality hazard ratio and the population for age group i. The per capita birth rate and per capita mortality rate for the total population m decline at constant fractional rates per year. Net migration for age group i is equal to I_i_-e_i P_i, where I_i is age-specific immigration per year and e_i is the age-specific per capita emigration rate per year.

Figure 2. Structure of the population sector

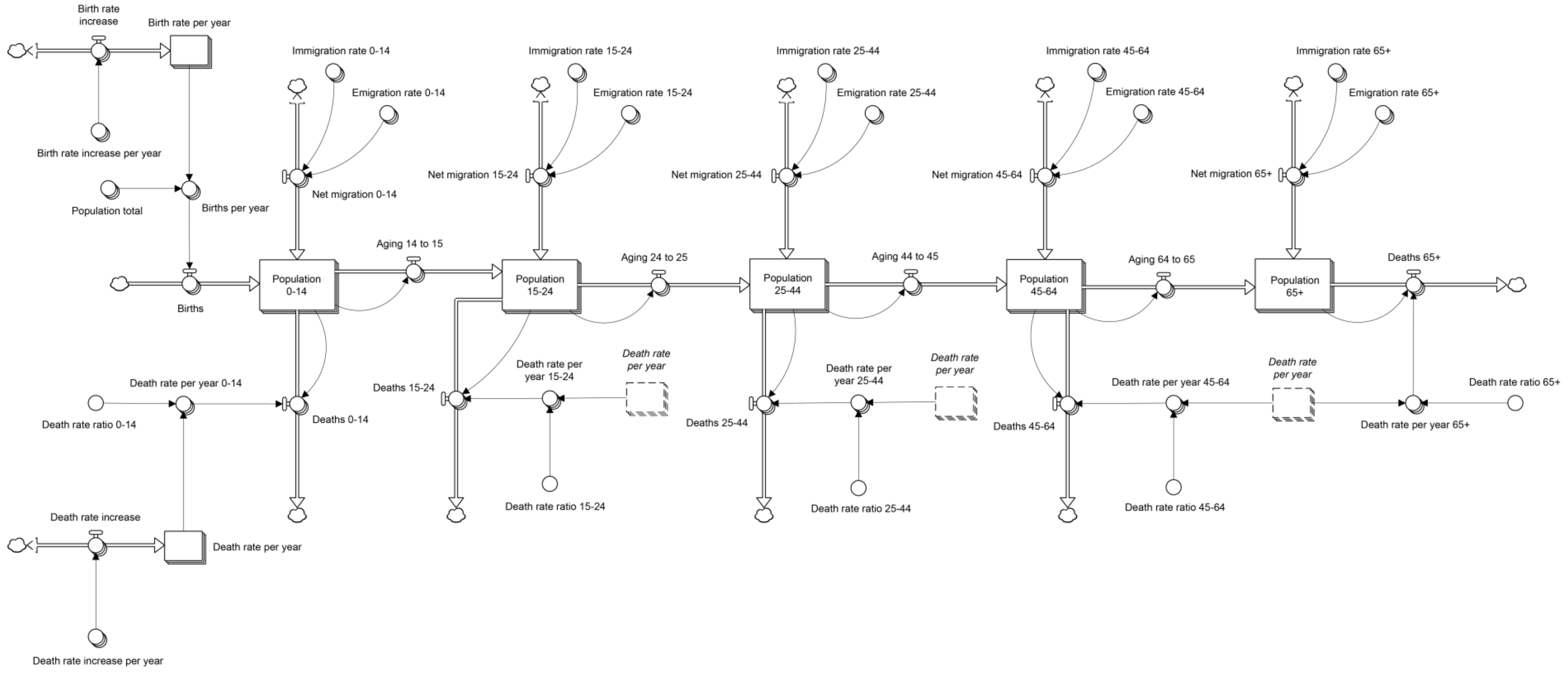

c) Psychological distress sector

Figure 3 shows the structure of the psychological distress sector, which models flows between states of low psychological distress (Kessler 10 [K10] scores 10−15) and moderate to very high psychological distress (K10 scores ≥ 16) in each age group. Numbers of people currently experiencing moderate to very high levels of psychological distress are modelled as stocks with inflows corresponding to psychological distress incidence and outflows corresponding to recovery.

Psychological distress incidence is equal to bhL, where b is the base per capita rate of distress onset per year, h is the product of the effects of developmental vulnerability during childhood, unemployment or (for people aged 15−24 years) non-participation in education or employment, and underemployment on psychological distress onset, and L is the number of people experiencing low levels of psychological distress. Moderately to highly distressed people in each age group recover at a yearly rate rH+T, where r is the per capita spontaneous recovery rate per year, H is the number of people currently experiencing moderate to very high psychological distress, and T is the number of people moving from a state of moderate to very high psychological distress to a state of low psychological distress per year due to effective mental health services. Aging of people experiencing moderate to very high levels of distress is modelled using a first-order delay like the population sector.

Figure 3. Structure of the psychological distress sector

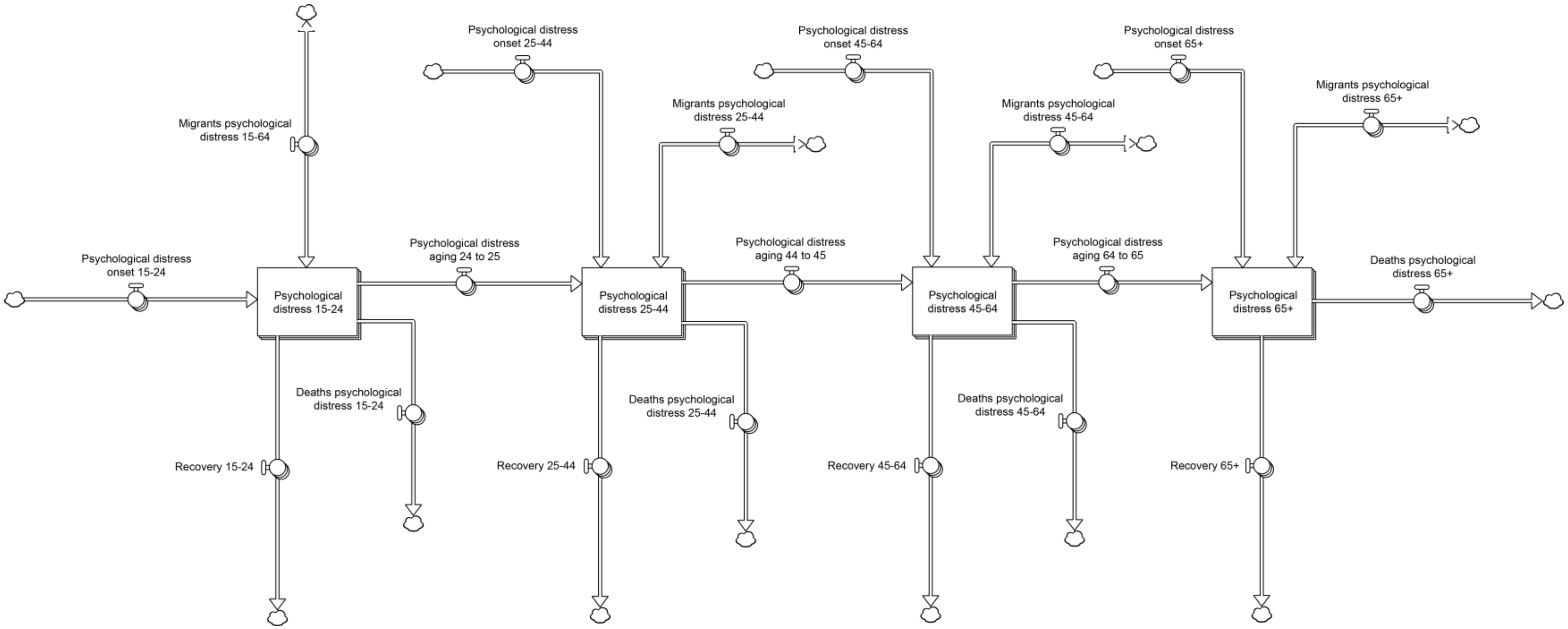

d) Developmental vulnerability sector

The developmental vulnerability sector (Figure 4) models the effect of exposure to adversity during childhood on the risk of developing mental disorders in adolescence and adulthood. The number of developmentally vulnerable children aged 0−14 years is modelled as a stock that grows as children at low risk of psychopathology transition to a state of higher risk. The model assumes that the onset of psychopathological vulnerability depends on cumulative exposure to adverse experiences, such as (e.g., parental psychological distress, physical and sexual abuse, domestic violence, poverty) and is irreversible.

The incidence of significant psychopathological vulnerability is equal to bpL, where b is the base per capita rate at which children at low risk of psychopathology transition to a state of higher risk per year, p is the effect of parental psychological distress on the risk of developing mental disorders in later life, and L is the number of low-risk 0−14-year-olds in the population.

Figure 4. Structure of the developmental vulnerability sector

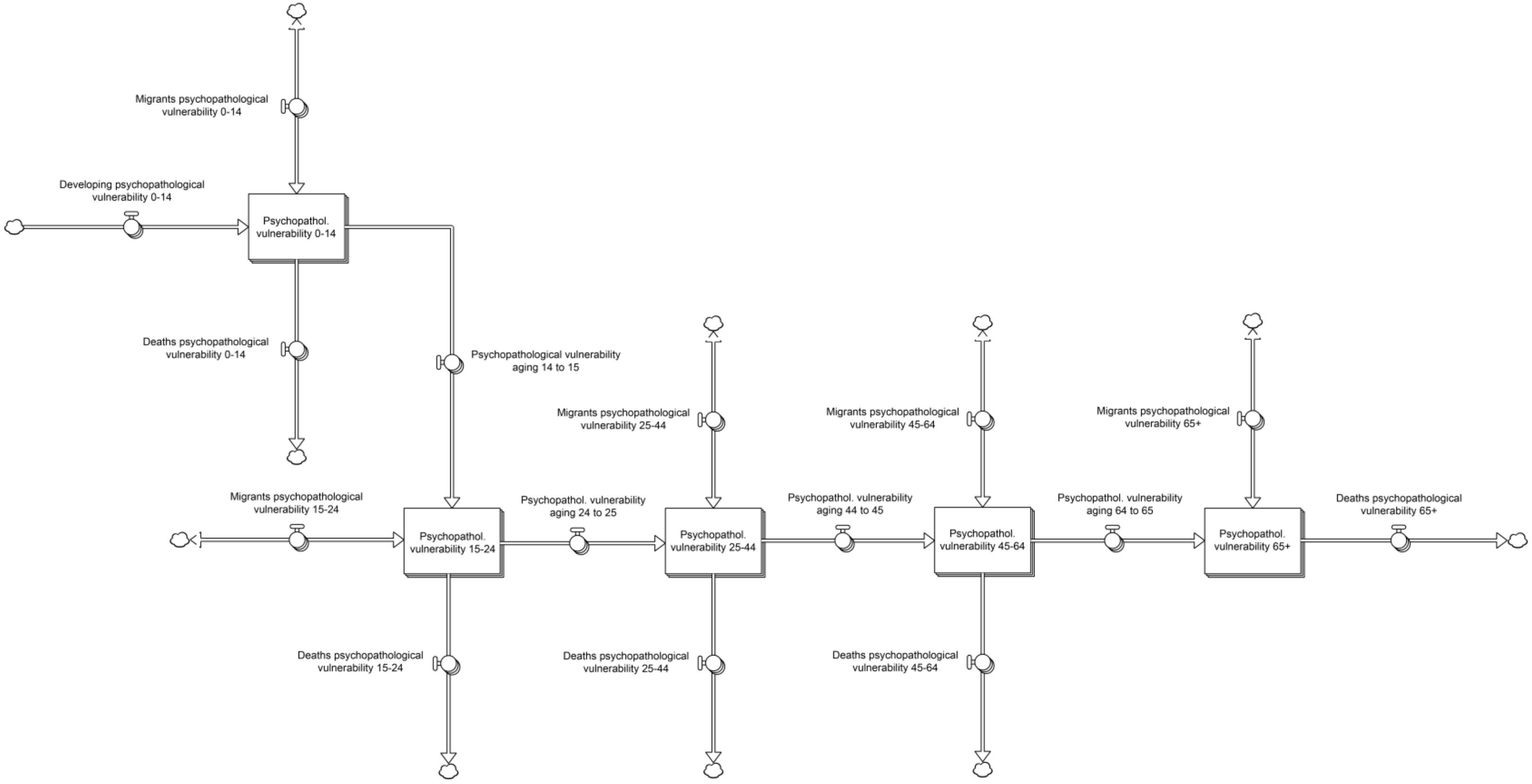

e) Suicidal behaviour sector

The suicidal behaviour sector, represented in Figure 5, models captures self-harm hospitalisations and suicide deaths. Note that we equate suicide attempts with intentional self-harm hospital admissions due to data availability constraints. Age-specific suicide attempt rates are calculated as $s_i L_i + r S_i H_i$, where L_i and H_i are the numbers of people in age group i experiencing low psychological distress and moderate to very high psychological distress, respectively, S_i is the per capita suicide attempt rate for mildly distressed people in age group i, and r is the suicide attempt rate ratio.

The number of suicide deaths per year is calculated as aF, where a is the suicide attempt rate and F is the suicide attempt lethality, that is the proportion of suicide attempts that are fatal.

Figure 5. Structure of the suicidal behaviour sector

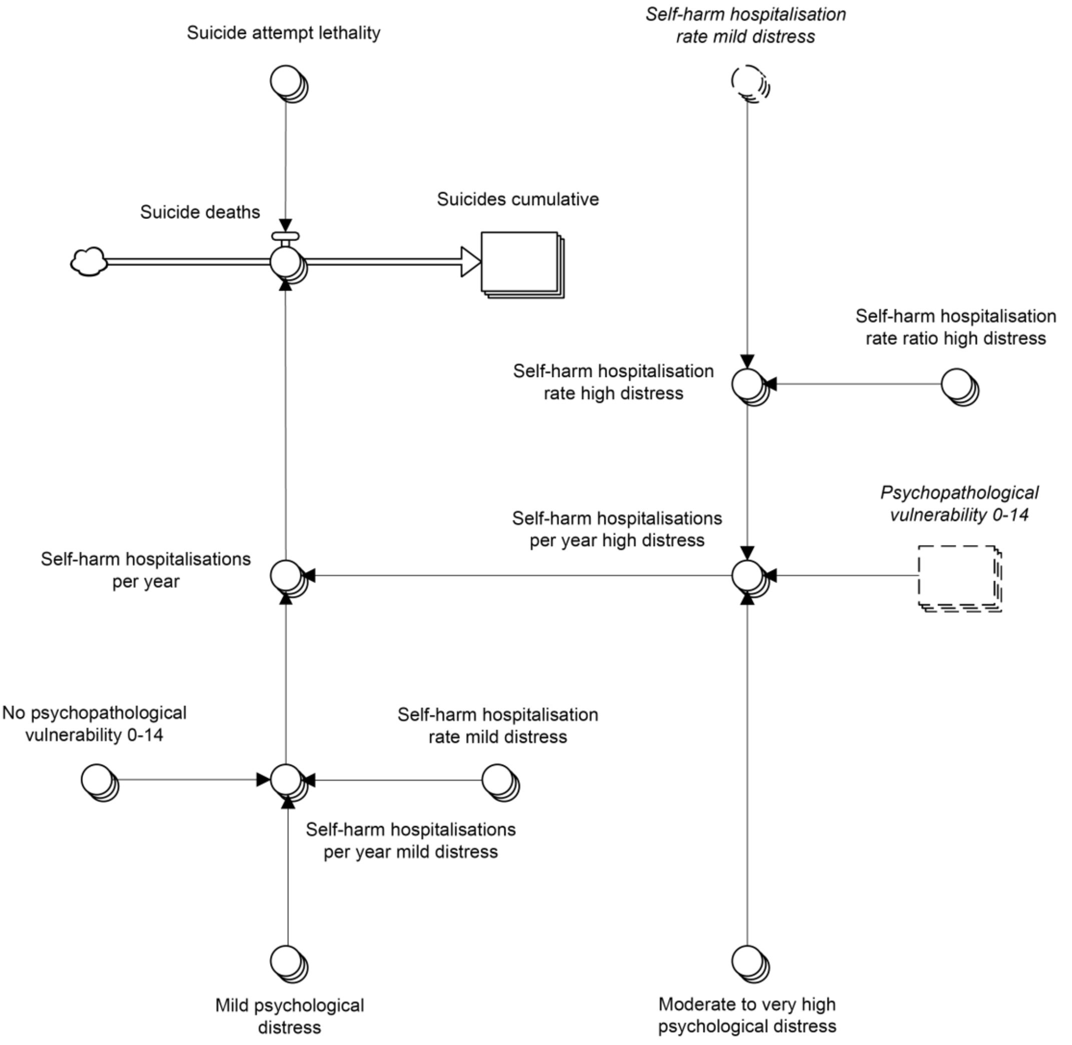

f) Education sector

Figure 6 represents the structure of the education sector, which captures post-secondary education and vocational training enrolment and completion rates (certificate III level and above) for people aged 15−64 years. Numbers of people currently studying for a post-secondary qualification are modelled as stocks with inflows corresponding to enrolment and outflows corresponding to completion and discontinuation (drop out prior to completion). Age-specific enrolment rates are calculated as bdN, where b is the base per capita enrolment rate per year, d is the effect of psychological distress on entry into post-secondary study, and N is the number of people not currently studying. Completion and discontinuation rates are equal to cS and hdS, respectively, where c is the per capita completion rate per year, d is the base per capita discontinuation rate per year, h is the effect of psychological distress on the discontinuation rate, and S is the number of people currently studying for a post-secondary qualification

Figure 6. Structure of the education sector

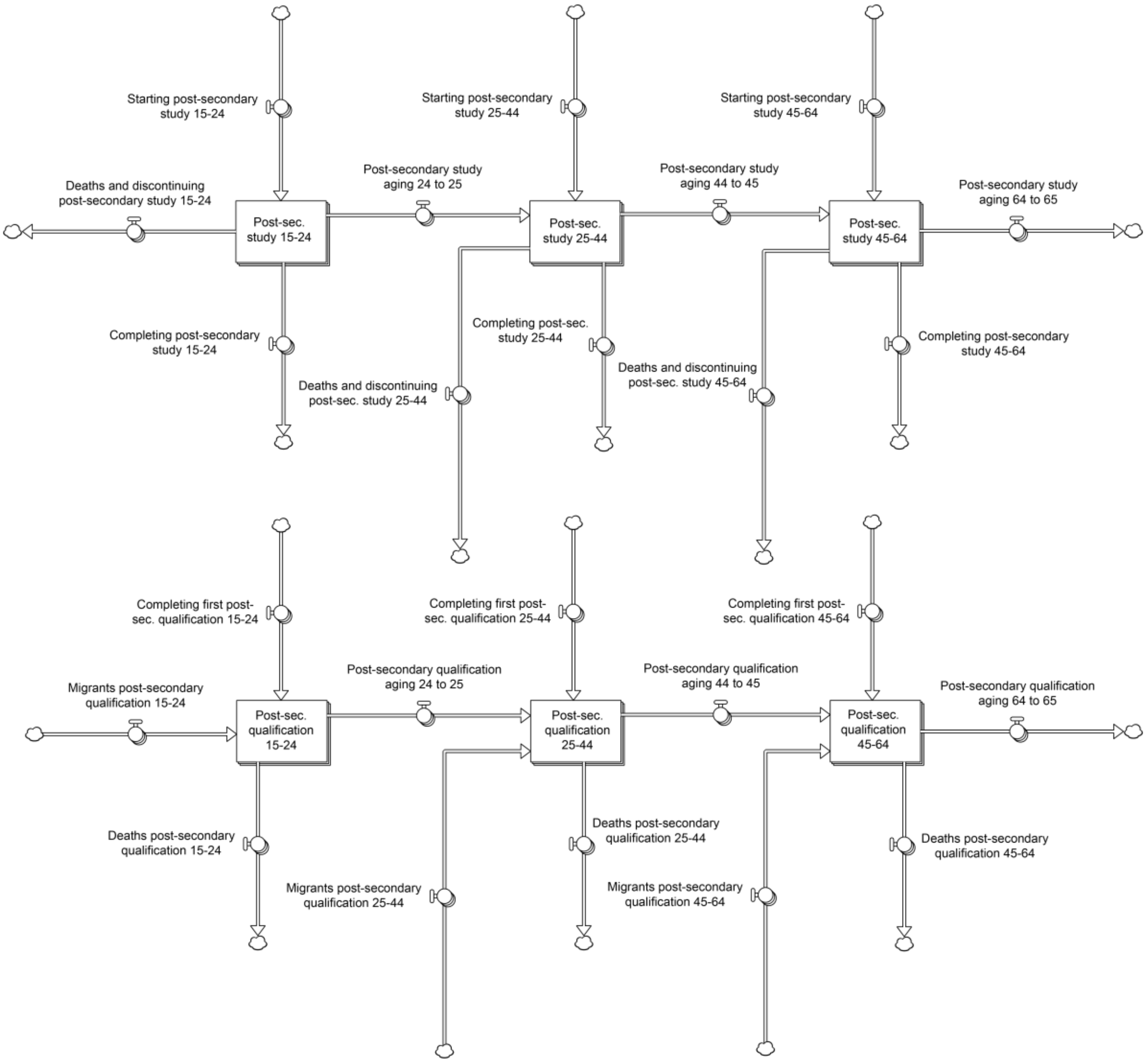

g) Employment sector

Figure 7 presents the employment sector, which models labour market transitions in the working-age population (15−64 years). The labour force is represented as nine stocks, corresponding to the numbers of fully employed, underemployed, and unemployed people aged 15−24 years, 25−44 years, and 45−64 years. When turning 15, adolescents are assumed to enter the population of those not in the labour force (NILF), which corresponds to people who are neither employed nor seeking employment. People not in the labour force who decide to seek employment enter the stocks of unemployed people, while those seeking employment (the unemployed) may leave the labour force. For each age range, net flows from the unemployed population to the population of people not in the labour force are calculated as fbU-hrN, where U and N are, respectively, the numbers of unemployed people and people not in the labour force, b is the base per capita rate that unemployed people leave the labour force per year, f is the effect of the unemployment rate on labour force participation (assumed to be greater than 1, so that increases in the unemployment rate reduce participation, r is the base per capita rate that people enter the labour force per year, and h is the product of the effects of psychological distress and completion of post-secondary education or vocational training on the labour force entry rate.

Figure 7. Structure of the employment sector

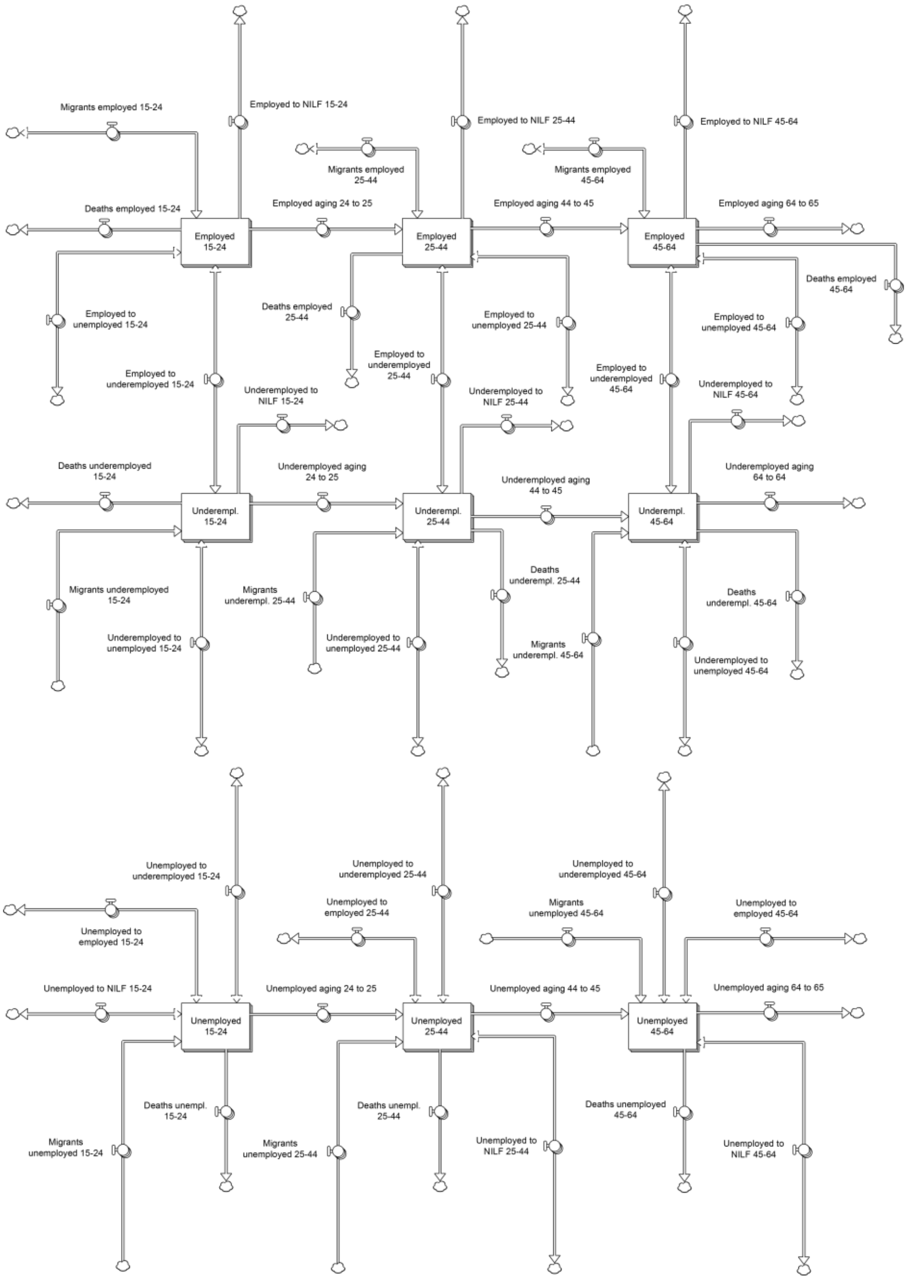

h) Mental health services sector

A high-level view of the mental health services sector is presented in Figure 8. This sector models the movement of people through the mental health care system. People with low or moderate to very high psychological distress engage with mental health services in two ways: they may either perceive a need for mental health care and seek help (for example from a general practitioner or online services), or they may present to an emergency department (ED) (for example for self-harm) without having previously perceived a need for treatment.

After engaging with mental health services, people may:

- recover following treatment, returning to the general population of people with low psychological distress and no perceived need for care,
- be treated but not recover, or
- disengage due to excessive waiting times, as a result of insufficient service capacity, or because they are dissatisfied with the care they receive.

People who are treated but do not recover return to perceiving a need for services and will eventually seek help again if they do not recover spontaneously; thus, people entering the mental health care system continue receiving treatment until they recover, disengage, or die (captured in the model, but not shown in figure 8).

Figure 8. High level view of the mental health services sector

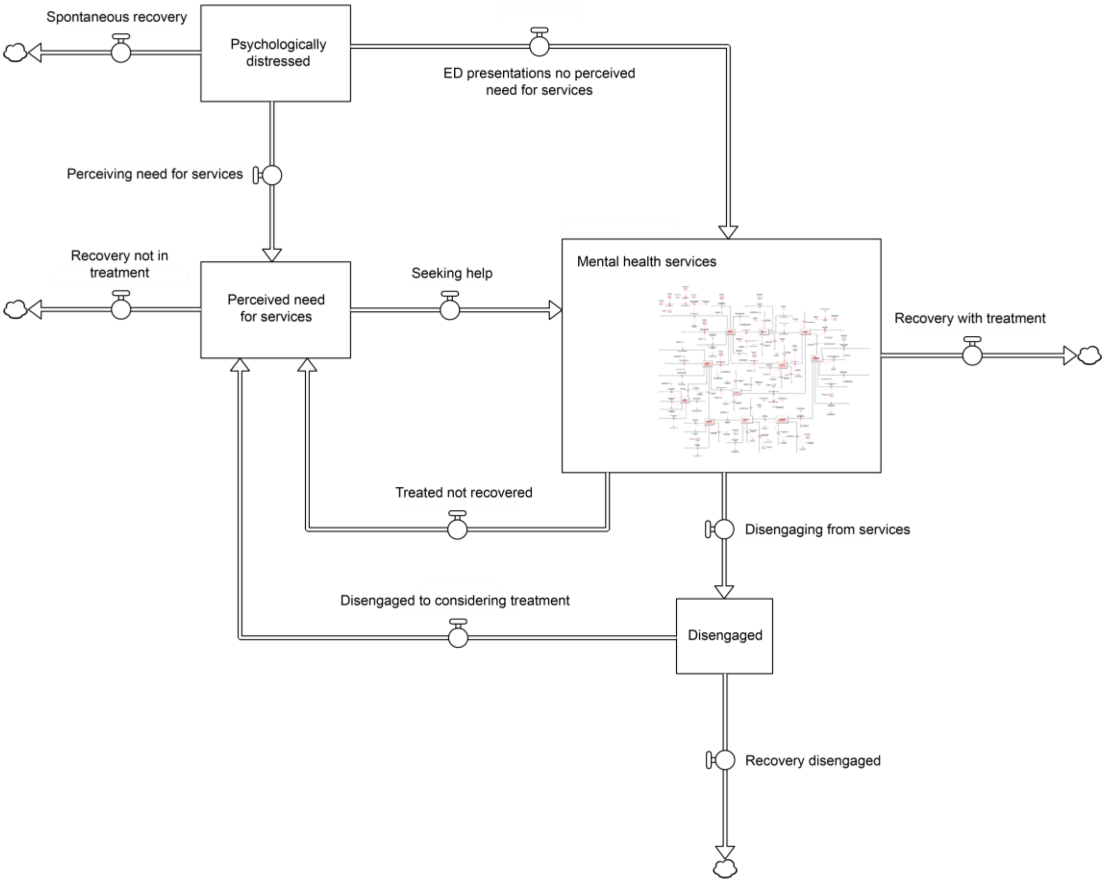

People with low or moderate to very high psychological distress who are not currently considering engaging with mental health services perceive a need for care at rates equal to Pi Di, where Pi is the per capita rate that people with distress level i perceive a need for care per year, and Di is the number of people with distress level i not currently considering treatment. The per capita rates Pi are assumed to increase at a constant rate per year due to increasing public awareness of high-prevalence mental disorders and available treatment options.

After perceiving a need for treatment, people engage with mental health services at per capita rates dependent on their age and levels of psychological distress. Recently treated patients who have not recovered

or disengaged from services return to perceiving a need for care and may attend subsequent appointments with a general practitioner (GP) or community mental health care (CMHC) psychiatric services (i.e., psychologists, psychiatrists and allied health services, hospital outpatient services), be admitted to a general or private hospital, commence online treatment, or present to an emergency department (Figure 9). Prospective and current patients may age, recover spontaneously, or transition from a state of low psychological distress to a state of moderate to very high distress.

Figure 9. Flow structure of the help-seeking, general practitioner services, and online services components of the mental health services sector

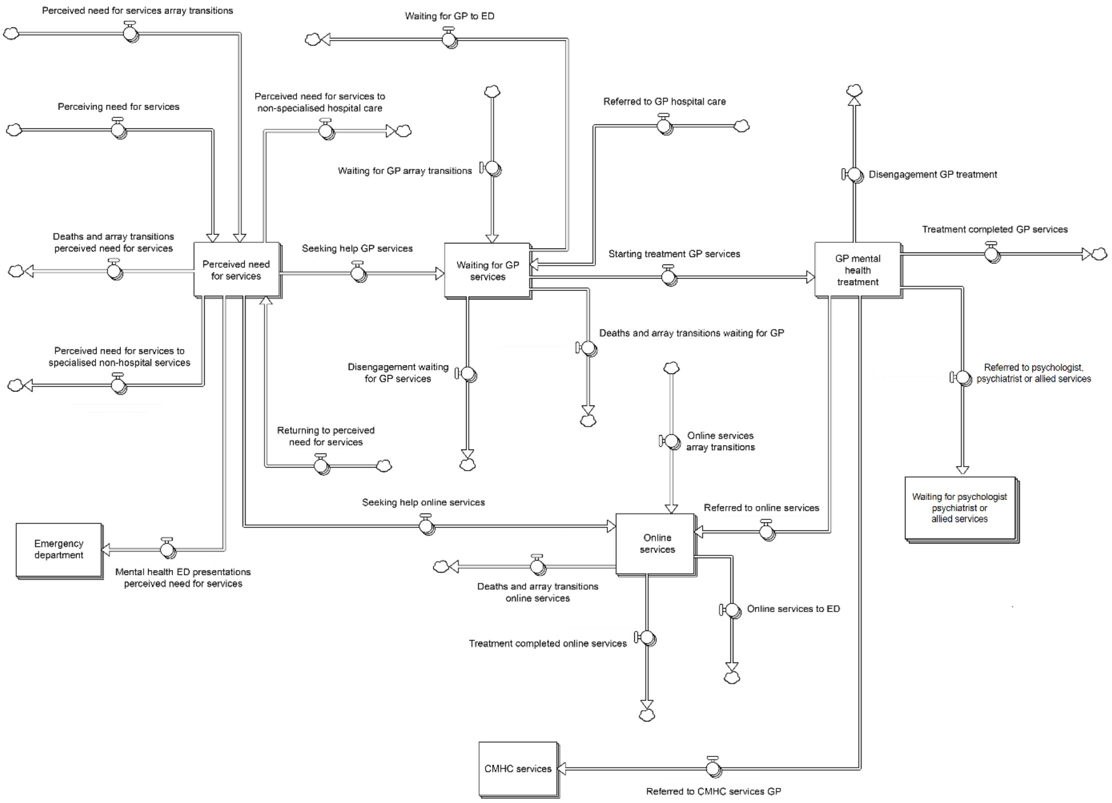

Figure 10 presents the structure of the psychologist, psychiatrist and allied health services component of the mental health services sector. Prior to receiving treatment, people referred to a psychologist, psychiatrist or allied health services by a general practitioner or after completing hospital inpatient care wait for a period of time that depends on service capacity and the total number of people waiting for care. The stock of people waiting for treatment also contains people currently engaged with specialised services who have planned (follow-up) appointments (these patients referred to services after receiving hospital care enter via the flow called 'Additional psychologist, psychiatrist or allied health services' in Figure 10). Service capacity, i.e., the number of psychologist, psychiatrist and allied health services that can be provided per year, increases at a constant rate per year, estimated from MBS claims data. People receiving treatment are referred to psychiatric hospital services, disengage from the mental health services system due to dissatisfaction with the care received, recover, or return to perceiving a need for care (these people flow back into the arrayed stock labelled 'Perceived need for services'.

Figure 10. Flow structure of the psychologist, psychiatrist and allied health services component of the mental health services sector

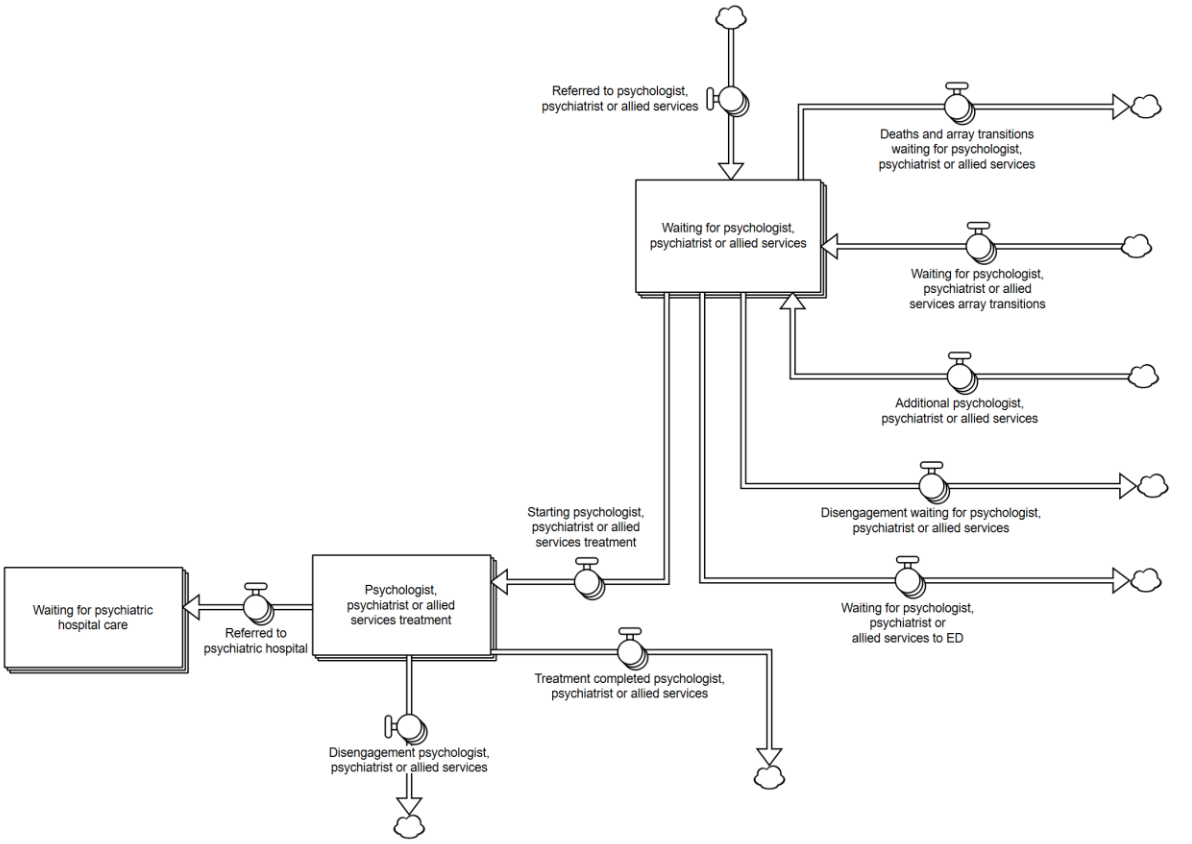

The Direct Access program is implemented by diverting part of the outflow linking the stock 'Perceived need for services' to 'Waiting for GP mental health services', and redirecting this flow to the stock 'Waiting for psychologist, psychiatrist or allied services'

i) COVID-19 modelling

The large impacts of the continuing COVID-19 pandemic were modelled as abrupt change in multiple flows directly affected by infection control measures (lockdowns, social distancing, international and interstate travel restrictions, including:

1. A decrease in the number of people arriving from overseas per year;

2. Increases in the per capita rates at which people transition from employment (including underemployment) to unemployment and from full employment to underemployment;

3. Reductions in per capita rates of non-acute mental health services provision (including general practitioner services, psychologist, psychiatrist and allied health services, public hospital outpatient services, and private mental health services);

4. An increase in the incidence of moderate to very high psychological distress resulting from social dislocation unrelated to job loss (e.g., working from home, not participating in recreational activities, restricted social gatherings) and anxiety about potential unemployment

To take into account the resilience of the population as people adapted to the new situation and resumed employment, we allow the per capita spontaneous recovery rate to increase as the prevalence of psychological distress increases above that observed immediately prior to the start of the pandemic. As the direct social and economic effects of the pandemic abate, the incidence of moderate to very high psychological distress declines, and the higher per capita recovery rate results in a relatively rapid decrease in distress prevalence, consistent with the empirical data.

Table 1. Parameters and data sources

Input	Stratification	Value	Notes
**Population**			
Birth rate increase per year	Major cities areas	-0.000179524501793	Estimated via constrained optimisation
	Regional and remote areas	0.0000116844462207	Estimated via constrained optimisation
Birth rate per year initial	Major cities areas	0.0141534960222	Estimated via constrained optimisation
	Regional and remote areas	0.0110538868806	Estimated via constrained optimisation
Death rate increase per year	Major cities areas	-0.0000820511739199	Estimated via constrained optimisation
	Regional and remote areas	-0.0000537083969755	Estimated via constrained optimisation
Death rate per year initial	Major cities areas	0.0061690886832	Estimated via constrained optimisation
	Regional and remote areas	0.00649754003436	Estimated via constrained optimisation
Death rate ratio	Age 0-14 years	0.0534891765279	Estimated via constrained optimisation
	Age 15-24 years	0.0558087629892	Estimated via constrained optimisation
	Age 25-44 years	0.120688852506	Estimated via constrained optimisation
	Age 45-64 years	0.612994223057	Estimated via constrained optimisation
	Age 65 years and over	6.23141665116	Estimated via constrained optimisation
Internal arrivals	Age 0-14 years, Major cities areas	212.990414923	Estimated via constrained optimisation
	Age 0-14 years, Regional and remote areas	329.380163965	Estimated via constrained optimisation
	Age 15-24 years, Major cities areas	257.453036692	Estimated via constrained optimisation
	Age 15-24 years, Regional and remote areas	278.448775713	Estimated via constrained optimisation
	Age 25-44 years, Major cities areas	549.839854026	Estimated via constrained optimisation
	Age 25-44 years, Regional and remote areas	577.066909172	Estimated via constrained optimisation
	Age 45-64 years, Major cities areas	176.102476024	Estimated via constrained optimisation
	Age 45-64 years, Regional and remote areas	318.891285201	Estimated via constrained optimisation
	Age 65 years and over, Major cities areas	71.1278729917	Estimated via constrained optimisation
	Age 65 years and over, Regional and remote areas	92.7549238018	Estimated via constrained optimisation
Pre-COVID-19 overseas arrivals	Age 0-14 years, Major cities areas	367.920275151	Estimated via constrained optimisation
	Age 0-14 years, Regional and remote areas	79.9119656052	Estimated via constrained optimisation
	Age 15-24 years, Major cities areas	379.9223432	Estimated via constrained optimisation
	Age 15-24 years, Regional and remote areas	304.826026614	Estimated via constrained optimisation
	Age 25-44 years, Major cities areas	857.572781349	Estimated via constrained optimisation
	Age 25-44 years, Regional and remote areas	762.366278789	Estimated via constrained optimisation
	Age 45-64 years, Major cities areas	252.361598189	Estimated via constrained optimisation
	Age 45-64 years, Regional and remote areas	84.8691430124	Estimated via constrained optimisation
	Age 65 years and over, Major cities areas	61.8087972239	Estimated via constrained optimisation
	Age 65 years and over, Regional and remote areas	22.2969647147	Estimated via constrained optimisation
Internal departure rate	Age 0-14 years, Major cities areas	0.0149638950265	Estimated via constrained optimisation
	Age 0-14 years, Regional and remote areas	0.0362455766574	Estimated via constrained optimisation
	Age 15-24 years, Major cities areas	0.0177957171053	Estimated via constrained optimisation
	Age 15-24 years, Regional and remote areas	0.0871589371532	Estimated via constrained optimisation
	Age 25-44 years, Major cities areas	0.0196433170939	Estimated via constrained optimisation

Input	Stratification	Value	Notes
	Age 25-44 years, Regional and remote areas	0.0642187766288	Estimated via constrained optimisation
	Age 45-64 years, Major cities areas	0.0106688267635	Estimated via constrained optimisation
	Age 45-64 years, Regional and remote areas	0.020581357867	Estimated via constrained optimisation
	Age 65 years and over, Major cities areas	0.0241480778842	Estimated via constrained optimisation
	Age 65 years and over, Regional and remote areas	0.0134933462649	Estimated via constrained optimisation
Overseas departure rate	Age 0-14 years, Major cities areas	0.00591542752402	Estimated via constrained optimisation
	Age 0-14 years, Regional and remote areas	0.0128522239546	Estimated via constrained optimisation
	Age 15-24 years, Major cities areas	0.006370667553	Estimated via constrained optimisation
	Age 15-24 years, Regional and remote areas	0.0604122588869	Estimated via constrained optimisation
	Age 25-44 years, Major cities areas	0.00137618674457	Estimated via constrained optimisation
	Age 25-44 years, Regional and remote areas	0.101391534562	Estimated via constrained optimisation
	Age 45-64 years, Major cities areas	0.00667081771369	Estimated via constrained optimisation
	Age 45-64 years, Regional and remote areas	0.00385910374906	Estimated via constrained optimisation
	Age 65 years and over, Major cities areas	0.00471586986143	Estimated via constrained optimisation
	Age 65 years and over, Regional and remote areas	0.00185354015205	Estimated via constrained optimisation
Population initial	Age 0-14 years, Major cities areas	997351.1229	Derived from Australian Bureau of Statistics, Estimated Resident Population by SA2 by Sex and Age (13)
	Age 0-14 years, Regional and remote areas	366708.8771	Derived from Australian Bureau of Statistics, Estimated Resident Population by SA2 by Sex and Age (13)
	Age 15-24 years, Major cities areas	732891.8674	Derived from Australian Bureau of Statistics, Estimated Resident Population by SA2 by Sex and Age (13)
	Age 15-24 years, Regional and remote areas	229185.1326	Derived from Australian Bureau of Statistics, Estimated Resident Population by SA2 by Sex and Age (13)
	Age 25-44 years, Major cities areas	1602355.991	Derived from Australian Bureau of Statistics, Estimated Resident Population by SA2 by Sex and Age (13)
	Age 25-44 years, Regional and remote areas	417608.5086	Derived from Australian Bureau of Statistics, Estimated Resident Population by SA2 by Sex and Age (13)
	Age 45-64 years, Major cities areas	1287201.178	Derived from Australian Bureau of Statistics, Estimated Resident Population by SA2 by Sex and Age (13)
	Age 45-64 years, Regional and remote areas	515687.8216	Derived from Australian Bureau of Statistics, Estimated Resident Population by SA2 by Sex and Age (13)
	Age 65 years and over, Major cities areas	700486.3139	Derived from Australian Bureau of Statistics, Estimated Resident Population by SA2 by Sex and Age (13)
	Age 65 years and over, Regional and remote areas	331933.6861	Derived from Australian Bureau of Statistics, Estimated Resident Population by SA2 by Sex and Age (13)
**Psychological distress**			
Psychological distress onset base rate	Age 15-24 years, Major cities areas	0.2268418273953	Estimated via constrained optimisation
	Age 15-24 years, Regional and remote areas	0.2159886805297	Estimated via constrained optimisation
	Age 25-44 years, Major cities areas	0.121475095144	Estimated via constrained optimisation
	Age 25-44 years, Regional and remote areas	0.117360243244	Estimated via constrained optimisation
	Age 45-64 years, Major cities areas	0.0959844619404	Estimated via constrained optimisation
	Age 45-64 years, Regional and remote areas	0.0875383956363	Estimated via constrained optimisation
	Age 65 years and over, Major cities areas	0.06251646846741	Estimated via constrained optimisation

Input	Stratification	Value	Notes
	Age 65 years and over, Regional and remote areas	0.0619756273436	Estimated via constrained optimisation
Psychological distress prevalence initial	Age 15-24 years, Major cities areas	0.245475105888	Estimated via constrained optimisation
	Age 15-24 years, Regional and remote areas	0.245475105888	Estimated via constrained optimisation
	Age 25-44 years, Major cities areas	0.206384301652	Estimated via constrained optimisation
	Age 25-44 years, Regional and remote areas	0.20641086408	Estimated via constrained optimisation
	Age 45-64 years, Major cities areas	0.211840187303	Estimated via constrained optimisation
	Age 45-64 years, Regional and remote areas	0.211999435254	Estimated via constrained optimisation
	Age 65 years and over, Major cities areas	0.18786617846225	Estimated via constrained optimisation
	Age 65 years and over, Regional and remote areas	0.18826169297355	Estimated via constrained optimisation
Effect of unemployment on psychological distress		1.732936000	Derived from Australian Bureau of Statistics (2012, Information paper. Use of the Kessler psychological distress scale in ABS health surveys, Australia, 2007-08. Cat. no. 4817.0.55.001. Australian Bureau of Statistics, Canberra) (14)
Unemployment rate ratio non-distressed		0.696177000	Derived from Australian Bureau of Statistics (2012, Information paper. Use of the Kessler psychological distress scale in ABS health surveys, Australia, 2007-08. Cat. no. 4817.0.55.001. Australian Bureau of Statistics, Canberra) (14)
Effect of underemployment on psychological distress		1.132448000	Derived from Dooley et al. (2000, J. Health Soc. Behav. 41, 421-436) (15)
Underemployment rate ratio non-distressed		0.975887900	Derived from Dooley et al. (2000, J. Health Soc. Behav. 41, 421-436) (15)
Effect of psychopathological vulnerability on distress onset		1.95	Derived from Green et al. (2019, Aust. N. Z. J. Psychiatry 53, 304-315)
Psychopathological vulnerability prevalence ratio non-distressed		0.918356100	Derived from Green et al. (2019, Aust. N. Z. J. Psychiatry 53, 304-315) (16)
Migrant psychological distress prevalence ratio		0.798851123	Australian Bureau of Statistics (2018, National Health Survey: first results, 2017-18. Cat. no. 4364.0.55.001. Australian Bureau of Statistics, Canberra) (17)
**Psychopathological vulnerability**			
Developing psychopathological vulnerability base rate	Age 0-14 years, Major cities areas	0.009010741	Estimated via constrained optimisation
	Age 0-14 years, Regional and remote areas	0.007094532	Estimated via constrained optimisation
Psychopathological vulnerability prevalence initial	Major cities areas	0.094140212	Estimated via constrained optimisation
	Regional and remote areas	0.093142710	Estimated via constrained optimisation
Effect of parental psychological distress on vulnerability	Age 0-14 years	1.63	Derived from Dean et al. (2018, Psychol. Med. 48, 2257-2263) (18)
Parental psychological distress prevalence ratio not vulnerable	Age 0-14 years	0.947830700	Derived from Dean et al. (2018, Psychol. Med. 48, 2257-2263) (18)
Proportion of population with dependent children	Age 15-24 years	0.043867662	Derived from Australian Bureau of Statistics. Family Characteristics and Transitions, Australia, 2012-13 data (19)

Input	Stratification	Value	Notes
	Age 25-44 years	0.542670616	Derived from Australian Bureau of Statistics. Family Characteristics and Transitions, Australia, 2012-13 data (19)
	Age 45-64 years	0.486845213	Derived from Australian Bureau of Statistics. Family Characteristics and Transitions, Australia, 2012-13 data (19)
	Age 65 years and over	0.178025686	Derived from Australian Bureau of Statistics. Family Characteristics and Transitions, Australia, 2012-13 data (19)
**Education**			
Discontinuing post-secondary study base rate	Age 15-24 years	0.425702620593	Estimated via constrained optimisation
	Age 25-44 years	0.124511971959	Estimated via constrained optimisation
	Age 45-64 years	0.827445477634	Estimated via constrained optimisation
Proportion completing first post-secondary qualification	Age 15-24 years, Major cities areas	0.361767605358	Estimated via constrained optimisation
	Age 15-24 years, Regional and remote areas	0.469019500822	Estimated via constrained optimisation
	Age 25-44 years, Major cities areas	0.199196059581	Estimated via constrained optimisation
	Age 25-44 years, Regional and remote areas	0.241229253477	Estimated via constrained optimisation
	Age 45-64 years, Major cities areas	0.227779529872	Estimated via constrained optimisation
	Age 45-64 years, Regional and remote areas	0.0675885557873	Estimated via constrained optimisation
Starting post-secondary study base rate	Age 15-24 years, Major cities areas	0.580231430123	Estimated via constrained optimisation
	Age 15-24 years, Regional and remote areas	0.318157468861	Estimated via constrained optimisation
	Age 25-44 years, Major cities areas	0.0780270176325	Estimated via constrained optimisation
	Age 25-44 years, Regional and remote areas	0.0940895604205	Estimated via constrained optimisation
	Age 45-64 years, Major cities areas	0.0488736923034	Estimated via constrained optimisation
	Age 45-64 years, Regional and remote areas	0.0678054879183	Estimated via constrained optimisation
Post-secondary study proportion initial	Age 15-24 years, Major cities areas	0.355115423	Derived from Australian Bureau of Statistics, Education and Work 2019 (20)
	Age 15-24 years, Regional and remote areas	0.202388926	Derived from Australian Bureau of Statistics, Education and Work 2019 (20)
	Age 25-44 years, Major cities areas	0.111649431	Derived from Australian Bureau of Statistics, Education and Work 2019 (20)
	Age 25-44 years, Regional and remote areas	0.096690348	Derived from Australian Bureau of Statistics, Education and Work 2019 (20)
	Age 45-64 years, Major cities areas	0.034433726	Derived from Australian Bureau of Statistics, Education and Work 2019 (20)
	Age 45-64 years, Regional and remote areas	0.039115740	Derived from Australian Bureau of Statistics, Education and Work 2019 (20)
Post-secondary qualification proportion initial	Age 15-24 years, Major cities areas	0.220696711	Derived from Australian Bureau of Statistics, Education and Work 2019 (20)
	Age 15-24 years, Regional and remote areas	0.182269013	Derived from Australian Bureau of Statistics, Education and Work 2019 (20)
	Age 25-44 years, Major cities areas	0.654297384	Derived from Australian Bureau of Statistics, Education and Work 2019 (20)

Input	Stratification	Value	Notes
	Age 25-44 years, Regional and remote areas	0.544850690	Derived from Australian Bureau of Statistics, Education and Work 2019 (20)
	Age 45-64 years, Major cities areas	0.538839012	Derived from Australian Bureau of Statistics, Education and Work 2019 (20)
	Age 45-64 years, Regional and remote areas	0.477588786	Derived from Australian Bureau of Statistics, Education and Work 2019 (20)
Death rate ratio post-secondary qualification		0.735294118	Derived from Backlund et al. (1999, Soc. Sci. Med. 49, 1373-1384) (21)
Migrant post-secondary qualification probability ratio		1.075613702	Derived from Australian Bureau of Statistics, Migrants, Education and Work 2019 (20)
Effect of psychological distress on post-secondary education		0.833333300	Derived from Lee et al. (2009, Br. J. Psychiatry 194, 411-417) (22)
Effect of psychological distress on discontinuation of post-secondary education		1.1	Derived from Lee et al. (2009, Br. J. Psychiatry 194, 411-417) (22)
**Employment**			
Employed to NILF base rate	Age 15-24 years	0.371573338306	Estimated via constrained optimisation
	Age 25-44 years	0.064365614564	Estimated via constrained optimisation
	Age 45-64 years	0.0569360434645	Estimated via constrained optimisation
Employed to underemployed base rate	Age 15-24 years	0.000984133452709	Estimated via constrained optimisation
	Age 25-44 years	0.306387391954	Estimated via constrained optimisation
	Age 45-64 years	0.337024197928	Estimated via constrained optimisation
Employed to unemployed base rate	Age 15-24 years	0.155600488688	Estimated via constrained optimisation
	Age 25-44 years	0.0539317451467	Estimated via constrained optimisation
	Age 45-64 years	0.042385725753	Estimated via constrained optimisation
NILF to unemployed base rate	Age 15-24 years, Major cities areas	0.728717263843	Estimated via constrained optimisation
	Age 15-24 years, Regional and remote areas	1.0502669821	Estimated via constrained optimisation
	Age 25-44 years, Major cities areas	0.458355491507	Estimated via constrained optimisation
	Age 25-44 years, Regional and remote areas	0.339806025419	Estimated via constrained optimisation
	Age 45-64 years, Major cities areas	0.148933300215	Estimated via constrained optimisation
	Age 45-64 years, Regional and remote areas	0.128669858682	Estimated via constrained optimisation
Underemployed to employed base rate	Age 15-24 years	2.08164756579	Estimated via constrained optimisation
	Age 25-44 years	3.51504895447	Estimated via constrained optimisation
	Age 45-64 years	4.18879222005	Estimated via constrained optimisation
Underemployed to NILF base rate	Age 15-24 years	0.00281146012383	Estimated via constrained optimisation
	Age 25-44 years	0.146844175522	Estimated via constrained optimisation
	Age 45-64 years	0.0725762580514	Estimated via constrained optimisation
Underemployed to unemployed base rate	Age 15-24 years	0.114968110716	Estimated via constrained optimisation
	Age 25-44 years	0.395113766934	Estimated via constrained optimisation
	Age 45-64 years	0.25634690096	Estimated via constrained optimisation
Unemployed to employed base rate	Age 15-24 years, Major cities areas	0.862505111761	Estimated via constrained optimisation
	Age 15-24 years, Regional and remote areas	0.931485378647	Estimated via constrained optimisation
	Age 25-44 years, Major cities areas	4.14264445603	Estimated via constrained optimisation

Input	Stratification	Value	Notes
	Age 25-44 years, Regional and remote areas	2.19530733234	Estimated via constrained optimisation
	Age 45-64 years, Major cities areas	2.6939372588	Estimated via constrained optimisation
	Age 45-64 years, Regional and remote areas	1.26200898964	Estimated via constrained optimisation
Unemployed to NILF base rate	Age 15-24 years	3.09737394771	Estimated via constrained optimisation
	Age 25-44 years	2.40348048622	Estimated via constrained optimisation
	Age 45-64 years	2.59226059947	Estimated via constrained optimisation
Unemployed to NILF rate coefficient unemployment	Age 15-24 years	1.26828920162	Estimated via constrained optimisation
	Age 25-44 years	1.17965648919	Estimated via constrained optimisation
	Age 45-64 years	1.0221490603	Estimated via constrained optimisation
Unemployed to underemployed base rate	Age 15-24 years, Major cities areas	4.05067465082	Estimated via constrained optimisation
	Age 15-24 years, Regional and remote areas	3.55278649145	Estimated via constrained optimisation
	Age 25-44 years, Major cities areas	0.253033373599	Estimated via constrained optimisation
	Age 25-44 years, Regional and remote areas	1.33599316279	Estimated via constrained optimisation
	Age 45-64 years, Major cities areas	0.272590449776	Estimated via constrained optimisation
	Age 45-64 years, Regional and remote areas	1.67048557039	Estimated via constrained optimisation
Employed proportion initial	Age 15-24 years, Major cities areas	0.616317913	Derived from Australian Bureau of Statistics, Labour Force, Australia 2019 (23)
	Age 15-24 years, Regional and remote areas	0.618544508	Derived from Australian Bureau of Statistics, Labour Force, Australia 2019 (23)
	Age 25-44 years, Major cities areas	0.790999868	Derived from Australian Bureau of Statistics, Labour Force, Australia 2019 (23)
	Age 25-44 years, Regional and remote areas	0.775968579	Derived from Australian Bureau of Statistics, Labour Force, Australia 2019 (23)
	Age 45-64 years, Major cities areas	0.713404939	Derived from Australian Bureau of Statistics, Labour Force, Australia 2019 (23)
	Age 45-64 years, Regional and remote areas	0.695142713	Derived from Australian Bureau of Statistics, Labour Force, Australia 2019 (23)
Unemployed proportion initial	Age 15-24 years, Major cities areas	0.087697142	Derived from Australian Bureau of Statistics, Labour Force, Australia 2019 (23)
	Age 15-24 years, Regional and remote areas	0.095630003	Derived from Australian Bureau of Statistics, Labour Force, Australia 2019 (23)
	Age 25-44 years, Major cities areas	0.033621655	Derived from Australian Bureau of Statistics, Labour Force, Australia 2019 (23)
	Age 25-44 years, Regional and remote areas	0.035541734	Derived from Australian Bureau of Statistics, Labour Force, Australia 2019 (23)
	Age 45-64 years, Major cities areas	0.024077597	Derived from Australian Bureau of Statistics, Labour Force, Australia 2019 (23)
	Age 45-64 years, Regional and remote areas	0.025003664	Derived from Australian Bureau of Statistics, Labour Force, Australia 2019 (23)
Underemployed proportion initial	Age 15-24 years	0.162401338	Derived from Australian Bureau of Statistics, Labour Force, Australia 2019 (23)
	Age 25-44 years	0.056678129	Derived from Australian Bureau of Statistics, Labour Force, Australia 2019 (23)

Input	Stratification	Value	Notes
	Age 45-64 years	0.058327686	Derived from Australian Bureau of Statistics, Labour Force, Australia 2019 (23)
Migrant underemployment probability ratio		0.862288655	Derived from Wilkins (2006, Aust. J. Labour Econ. 9, 371-393) (24)
Migrant unemployment probability ratio		0.994422716	Derived from Australian Bureau of Statistics, Labour Force Detailed, Australia 2019 (25)
Migrant employment probability ratio		0.956702207	Derived from Australian Bureau of Statistics, Labour Force Detailed, Australia 2019 (25)
Effect of psychological distress on participation		0.839659600	Derived from Frijters et al. (2014, Health Econ. 23, 1058–1071) (26)
Effect of psychological distress on employment		0.839659600	Derived from Frijters et al. (2014, Health Econ. 23, 1058–1071) (26)
Effect of post-secondary education on participation		1.351342518	Derived from Australian Bureau of Statistics (2020, Education and work, Australia, May 2020. Cat. no. 6227.0. Australian Bureau of Statistics, Canberra) (27)
Effect of post-secondary education on employment		1.046560897	Derived from Australian Bureau of Statistics (2020, Education and work, Australia, May 2020. Cat. no. 6227.0. Australian Bureau of Statistics, Canberra) (27)
Death rate ratio unemployed		1.22	Derived from Sorlie and Rogot (1990, Am. J. Epidemiol. 132, 983–992)
Post-secondary qualification probability ratio NILF		0.679195716	Derived from Australian Bureau of Statistics (2020, Education and work, Australia, May 2020. Cat. no. 6227.0. Australian Bureau of Statistics, Canberra) (27)
Post-secondary qualification probability ratio underemployed		0.862828720	Derived from Wilkins (2004, The extent and consequences of underemployment in Australia. Melbourne Institute working paper no. 16/04. (28)The University of Melbourne, Melbourne) and Wilkins (2006, Aust. J. Labour Econ. 9, 371-393)(29)
Post-secondary study employed proportion ratio	Age 15-24 years, Major cities areas	0.519269670	Estimated via constrained optimisation
	Age 15-24 years, Regional and remote areas	0.766553475	Estimated via constrained optimisation
Unemployed to NILF rate coefficient unemployment	Age 15-24 years	1.663855685	Estimated via constrained optimisation
	Age 25-44 years	1.148233134	Estimated via constrained optimisation
	Age 45-64 years	1.018995294	Estimated via constrained optimisation
Effect of education on underemployed to employed rate		1.407043821	Derived from Wilkins (2004, The extent and consequences of underemployment in Australia. Melbourne Institute working paper no. 16/04. (28)The University of Melbourne, Melbourne) and Wilkins (2006, Aust. J. Labour Econ. 9, 371-393) (29)
**Suicidal behaviour**			
Self-harm hospitalisation rate non-distressed	Age 0-14 years, Major cities areas	0.0000897859635462	Estimated via constrained optimisation
	Age 0-14 years, Regional and remote areas	0.000104422325439	Estimated via constrained optimisation
	Age 15-24 years, Major cities areas	0.000382301000684	Estimated via constrained optimisation
	Age 15-24 years, Regional and remote areas	0.000537238146414	Estimated via constrained optimisation

Input	Stratification	Value	Notes
	Age 25-44 years, Major cities areas	0.000256210492217	Estimated via constrained optimisation
	Age 25-44 years, Regional and remote areas	0.000441266923084	Estimated via constrained optimisation
	Age 45-64 years, Major cities areas	0.000229489079849	Estimated via constrained optimisation
	Age 45-64 years, Regional and remote areas	0.000246517210309	Estimated via constrained optimisation
	Age 65 years and over, Major cities areas	0.00011900362119	Estimated via constrained optimisation
	Age 65 years and over, Regional and remote areas	0.000100911845668	Estimated via constrained optimisation
Suicide attempt lethality initial	Age 0-14 years	0.0453389351676	Estimated via constrained optimisation
	Age 15-24 years	0.0453917818388	Estimated via constrained optimisation
	Age 25-44 years	0.106380716741	Estimated via constrained optimisation
	Age 45-64 years	0.159548995443	Estimated via constrained optimisation
	Age 65 years and over	0.288748120745	Estimated via constrained optimisation
Suicide attempt lethality multiplier increase per year		0.00407870628992	Estimated via constrained optimisation
**Services**			
CMHC service capacity increase per year	Major cities areas	1930.5167042	Estimated via constrained optimisation
	Regional and remote areas	34.1647793682	Estimated via constrained optimisation
CMHC service capacity initial	Major cities areas	30446.7548779	Estimated via constrained optimisation
	Regional and remote areas	15829.6133847	Estimated via constrained optimisation
General practitioner service capacity increase per year	Major cities areas	985.126027207	Estimated via constrained optimisation
	Regional and remote areas	306.995610283	Estimated via constrained optimisation
General practitioner service capacity initial	Major cities areas	9367.33895896	Estimated via constrained optimisation
	Regional and remote areas	3599.17123225	Estimated via constrained optimisation
Non-specialised hospital capacity increase per year	Major cities areas	8.93523781116	Estimated via constrained optimisation
	Regional and remote areas	-0.610045816449	Estimated via constrained optimisation
Non-specialised hospital capacity initial	Major cities areas	227.404310088	Estimated via constrained optimisation
	Regional and remote areas	143.451915818	Estimated via constrained optimisation
Private hospital capacity increase per year	Major cities areas	5.61792583709	Estimated via constrained optimisation
	Regional and remote areas	1.96947544058	Estimated via constrained optimisation
Private hospital capacity initial	Major cities areas	158.993058349	Estimated via constrained optimisation
	Regional and remote areas	65.0808754554	Estimated via constrained optimisation
Private outpatient service capacity increase per year	Major cities areas	39.0356940312	Estimated via constrained optimisation
	Regional and remote areas	11.7215046837	Estimated via constrained optimisation
Private outpatient service capacity initial	Major cities areas	859.153369475	Estimated via constrained optimisation
	Regional and remote areas	78.8343271457	Estimated via constrained optimisation
Psychiatric hospital capacity increase per year	Major cities areas	2.37269235343	Estimated via constrained optimisation
	Regional and remote areas	0.448916371637	Estimated via constrained optimisation
Psychiatric hospital capacity initial	Major cities areas	468.796715696	Estimated via constrained optimisation
	Regional and remote areas	173.334211282	Estimated via constrained optimisation
Psychiatrist and allied service capacity increase per year	Major cities areas	1230.78162489	Estimated via constrained optimisation
	Regional and remote areas	396.274719606	Estimated via constrained optimisation
Psychiatrist and allied service capacity initial	Major cities areas	25988.6395067	Estimated via constrained optimisation
	Regional and remote areas	6087.47300199	Estimated via constrained optimisation

Input	Stratification	Value	Notes
Mean treatment duration non-specialised hospital care		0.883845357	Derived from national data on mental health-related hospitalisations published by the Australian Institute of Health and Welfare (available at: https://www.aihw.gov.au/reports-data/health-welfare-services/mental-health-services/data) (11)
Effect of psychological distress on help seeking	High distress	0.950333300	Derived from Australian Bureau of Statistics (2012, Information paper. Use of the Kessler psychological distress scale in ABS health surveys, Australia, 2007-08. Cat. no. 4817.0.55.001. Australian Bureau of Statistics, Canberra) (14)
	Low distress	1	Reference category
Mean treatment duration online services		6	Derived from Christensen et al. (2004, Br. Med. J. 328, 265) (30)
Referral rate online services		0.046749000	Derived from national data on mental health-related general practitioner services published by the Australian Institute of Health and Welfare (available at: https://www.aihw.gov.au/reports-data/health-welfare-services/mental-health-services/data) (11)
Mean treatment duration private hospital		2.526374701	Derived from national data on mental health-related hospitalisations published by the Australian Institute of Health and Welfare (available at: https://www.aihw.gov.au/reports-data/health-welfare-services/mental-health-services/data) (11)
Effect of disengagement on recovery		0.454296830	Derived from Australian Bureau of Statistics (2012, Information paper. Use of the Kessler psychological distress scale in ABS health surveys, Australia, 2007-08. Cat. no. 4817.0.55.001. Australian Bureau of Statistics, Canberra) (14)
Effect of disengagement on psychological distress		2.201204000	Derived from Australian Bureau of Statistics (2012, Information paper. Use of the Kessler psychological distress scale in ABS health surveys, Australia, 2007-08. Cat. no. 4817.0.55.001. Australian Bureau of Statistics, Canberra) (14)
Disengaged to perceived need for services rate		5.181821348	Estimated via constrained optimisation
Disengagement rate waiting		0.2620284	Derived from Tyrer et al. (1995, Lancet 345, 756–759) (31)
Disengagement rate hospital care		0.051642558	Derived from state-level consumer survey data for 2016-17 published by the Australian Institute of Health and Welfare (available at: https://www.aihw.gov.au/reports-data/health-welfare-services/mental-health-services/data) (11)
Disengagement rate non-hospital care		0.03909747	Derived from state-level consumer survey data for 2016-17 published by the Australian Institute of Health and Welfare (available at: https://www.aihw.gov.au/reports-

Input	Stratification	Value	Notes
			data/health-welfare-services/mental-health-services/data) (11)
Natural recovery rate ratio low distress		3.225351000	Derived from Kessler et al. (1997, J. Affect. Disord. 45, 19-30) (32)
Effect of psychological distress on ED presentation rate	High distress	7.420126000	Derived from Australian Bureau of Statistics (2012, Information paper. Use of the Kessler psychological distress scale in ABS health surveys, Australia, 2007-08. Cat. no. 4817.0.55.001. Australian Bureau of Statistics, Canberra) (14)
	Low distress	1	Reference category
Post-discharge non-CMHC services referral proportion general practitioner		0.5	Assumes half of patients not referred to CMHC services after discharge from hospital care are referred to a general practitioner. The remaining patients (i.e., those not referred to CMHC services or a general practitioner) are referred to a psychiatrist or allied mental health professional.
Effect of psychological distress on non-specialised hospitalisation rate	High distress	7.420126000	Derived from Australian Bureau of Statistics (2012, Information paper. Use of the Kessler psychological distress scale in ABS health surveys, Australia, 2007-08. Cat. no. 4817.0.55.001. Australian Bureau of Statistics, Canberra) (14)
	Low distress	1	Reference category
Recovery base rate CMHC services		0.025450458	Per-service recovery rate derived from data on patient outcomes and numbers of services per patient per year published online by the Australian Institute of Health and Welfare (https://www.aihw.gov.au/reports-data/health-welfare-services/mental-health-services/data) (11)
Recovery rate online services	High distress	0.185074640	Derived from Christensen et al. (2004, Br. Med. J. 328, 265) (30) and Cuijpers et al. (2009, Br. J. Gen. Pract., doi: 10.3399/bjgp09X395139) (33)
	Low distress	0.400000000	Derived from Christensen et al. (2004, Br. Med. J. 328, 265) (30)
Psychological treatment rate general practitioner services		0.483438750	Derived from national data on mental health-related general practitioner services published by the Australian Institute of Health and Welfare (available at: https://www.aihw.gov.au/reports-data/health-welfare-services/mental-health-services/data) (11)
Recovery rate ratio general practitioner services	High distress	0.462686600	Derived from Cuijpers et al. (2009, Br. J. Gen. Pract., doi: 10.3399/bjgp09X395139) (33)
	Low distress	1	Reference category
CMHC services referral rate increase per year		0.085217551787	Estimated via constrained optimisation
CMHC services referral rate initial		0.527696517828	Estimated via constrained optimisation
Hospital admission rate increase per year		-0.012	Estimated via constrained optimisation
Hospital admission rate initial		0.38	Estimated via constrained optimisation

Input	Stratification	Value	Notes
Referral rate psychiatrist and allied services increase per year		0.00756445703239	Estimated via constrained optimisation
Referral rate psychiatrist or allied services initial		0.0690274038646	Estimated via constrained optimisation
Seeking help general practitioner services base rate age array	Age 0-14 years	0.383103263386	Estimated via constrained optimisation
	Age 15-24 years	1.23	Estimated via constrained optimisation
	Age 25-44 years	1.86979522671	Estimated via constrained optimisation
	Age 45-64 years	1.72896939943	Estimated via constrained optimisation
	Age 65 years and over	0.510801177351	Estimated via constrained optimisation
Seeking help general practitioner services rate increase per year		0.035	Estimated via constrained optimisation
Seeking help general practitioner services rate ratio regional	Major cities areas	1.000000000	Estimated via constrained optimisation
	Regional and remote areas	0.99	Estimated via constrained optimisation
Waiting for general practitioner mental health services total initial		50	Estimated via constrained optimisation
Disengaged to perceived need for services rate		4.87317230971	Estimated via constrained optimisation
Perceived need for services low distress proportion initial		0.454918373954	Estimated via constrained optimisation
Perceived need for services total initial		721186.88111	Estimated via constrained optimisation
Perceiving need for services base rate	High psychological distress	0.183950239614	Estimated via constrained optimisation
	Low psychological distress	0.0295569313105	Estimated via constrained optimisation
Perceiving need for services rate increase per year		0.00000382682545797	Estimated via constrained optimisation
Additional psychiatrist and allied services rate ratio regional	Major cities areas	1	
	Regional and remote areas	0.686576899029	Estimated via constrained optimisation
Additional psychiatrist or allied services rate age array	Age 0-14 years	1.55048997158	Estimated via constrained optimisation
	Age 15-24 years	3.45817669532	Estimated via constrained optimisation
	Age 25-44 years	4.65238044375	Estimated via constrained optimisation
	Age 45-64 years	4.85879278368	Estimated via constrained optimisation
	Age 65 years and over	1.04340949413	Estimated via constrained optimisation
Waiting for psychiatrist or allied services total initial		200000	Estimated via constrained optimisation
Additional CMHC service contacts rate age array	Age 0-14 years	1.34495017118	Estimated via constrained optimisation
	Age 15-24 years	4	Estimated via constrained optimisation
	Age 25-44 years	5.21103284954	Estimated via constrained optimisation
	Age 45-64 years	3.83018590037	Estimated via constrained optimisation
	Age 65 years and over	1.38251006276	Estimated via constrained optimisation
CMHC services referral rate ED		0.984363174627	Estimated via constrained optimisation
ED presentation base rate 65+	Major cities areas	0.00134351710877	Estimated via constrained optimisation
	Regional and remote areas	0.00300672463694	Estimated via constrained optimisation
ED presentation rate ratio age	Age 0-14 years	0.685873215504	Estimated via constrained optimisation
	Age 15-24 years	1.9	Estimated via constrained optimisation
	Age 25-44 years	2.17904190199	Estimated via constrained optimisation

Input	Stratification	Value	Notes
	Age 45-64 years	1.5180600426	Estimated via constrained optimisation
ED presentation rate ratio perceived need for services		2.0902785593	Estimated via constrained optimisation
Private hospital referral rate age array	Age 0-14 years	0.00112524185213	Estimated via constrained optimisation
	Age 15-24 years	0.0231849479888	Estimated via constrained optimisation
	Age 25-44 years	0.0324186208265	Estimated via constrained optimisation
	Age 45-64 years	0.027	Estimated via constrained optimisation
	Age 65 years and over	0.01204	Estimated via constrained optimisation
Private hospital referral rate ratio regional	Major cities areas	1	
	Regional and remote areas	1.06386266701	Estimated via constrained optimisation
Additional admission rate non-specialised hospital care age array	Age 0-14 years	0.00185	Estimated via constrained optimisation
	Age 15-24 years	0.00264623067307	Estimated via constrained optimisation
	Age 25-44 years	0.0042	Estimated via constrained optimisation
	Age 45-64 years	0.0042	Estimated via constrained optimisation
	Age 65 years and over	0.0069	Estimated via constrained optimisation
Additional non-specialised hospitalisations rate ratio regional	Major cities areas	1	
	Regional and remote areas	1.27437086407	Estimated via constrained optimisation
Psychiatric hospital admission proportion	Age 0-14 years	0.0943974042179	Estimated via constrained optimisation
	Age 15-24 years	0.63747609393	Estimated via constrained optimisation
	Age 25-44 years	0.69156658947	Estimated via constrained optimisation
	Age 45-64 years	0.659975647822	Estimated via constrained optimisation
	Age 65 years and over	0.372420713075	Estimated via constrained optimisation
Referred to psychiatric hospital rate	Age 0-14 years	0.000047867862711	Estimated via constrained optimisation
	Age 15-24 years	0.0115173099976	Estimated via constrained optimisation
	Age 25-44 years	0.00991773107653	Estimated via constrained optimisation
	Age 45-64 years	0.0102388433798	Estimated via constrained optimisation
	Age 65 years and over	0.0170967231675	Estimated via constrained optimisation
Private outpatient services referral rate age array	Age 0-14 years	0.000194688788667	Estimated via constrained optimisation
	Age 15-24 years	0.0715	Estimated via constrained optimisation
	Age 25-44 years	0.135194320831	Estimated via constrained optimisation
	Age 45-64 years	0.173403341797	Estimated via constrained optimisation
	Age 65 years and over	0.0539091156385	Estimated via constrained optimisation
Private outpatient services referral rate ratio regional	Major cities areas	1	
	Regional and remote areas	0.22	Estimated via constrained optimisation
**Impacts of COVID-19**			
Maximum decrease in overseas arrivals due to COVID-19		0.253602578529	Estimated via constrained optimisation
Overseas migration effect duration		5.68216709844	Estimated via constrained optimisation
Migration effect starting year		2020.167	Equivalent to March 2020
Employed to underemployed rate multiplier ratio	Age 15-24 years	9	Estimated via constrained optimisation
	Age 25-44 years	1.62896407287	Estimated via constrained optimisation
	Age 45-64 years	0.55	Estimated via constrained optimisation

Input	Stratification	Value	Notes
Unemployment effect decay rate		0.0562277521291	Estimated via constrained optimisation
Unemployment increase effect	Age 15-24 years, Major cities areas	4.09299349595	Estimated via constrained optimisation
	Age 15-24 years, Regional and remote areas	2	Estimated via constrained optimisation
	Age 25-44 years, Major cities areas	3.00525328032	Estimated via constrained optimisation
	Age 25-44 years, Regional and remote areas	2.19569554915	Estimated via constrained optimisation
	Age 45-64 years, Major cities areas	5.666875639	Estimated via constrained optimisation
	Age 45-64 years, Regional and remote areas	4	Estimated via constrained optimisation
Unemployment increase starting year		2020.167	Equivalent to March 2020
Years to increase unemployment		0.000505023620102	Estimated via constrained optimisation
Maximum decrease in services provision due to COVID-19		0.68	Estimated via constrained optimisation
Services effect starting year		2020.167	Equivalent to March 2020
Services effect duration		2.8	Estimated via constrained optimisation
Effect of doubling psychological distress prevalence on recovery		400.06566065	Estimated via constrained optimisation
Sense of Community Index decrease	Age 15-24 years	0.67	Estimated via constrained optimisation
	Age 25-44 years	0.27	Estimated via constrained optimisation
	Age 45-64 years	0.15	Estimated via constrained optimisation
	Age 65 years and over	-1	Estimated via constrained optimisation
Social connectedness decay rate		0.745789758102	Estimated via constrained optimisation
Social dislocation duration		0.324297202308	Estimated via constrained optimisation
Years to reach minimum Sense of Community Index		0.0609843483592	Estimated via constrained optimisation
Social dislocation starting year		2020.167	Equivalent to March 2020
Sense of Community Index initial		9.149557522	Derived from Handley et al. (2012, Soc. Psychiatry Psychiatr. Epidemiol. 47, 1281–1290) (34)
Effect of Sense of Community Index increase on distress		0.64	Derived from Handley et al. (2012, Soc. Psychiatry Psychiatr. Epidemiol. 47, 1281–1290) (34)
Effect of doubling unemployment on psychological distress		1.349859	Derived from Dooley et al., 1988. J. Soc. Issues 44, 107-123 (35)
Effect of doubling unemployment on unemployment effect		1.000000000	Derived from Dooley et al., 1988. J. Soc. Issues 44, 107-123 (35)
Post-secondary study proportion employed		0.678309365	Derived from Australian Bureau of Statistics (2020, Education and work, Australia, May 2020. Cat. no. 6227.0. Australian Bureau of Statistics, Canberra) (36)
Pre-intervention proportion discontinuing study unemployed		0.5	Assumes that half of post-secondary students becoming unemployed due to the COVID-19 pandemic will discontinue study
Pre-COVID-19 recovery base rate	Age 15-24 years	0.06833333	Derived from Jokela et al. (2011, J. Affect. Disord. 130, 454-461) (37)
	Age 25-44 years	0.06833333	Derived from Jokela et al. (2011, J. Affect. Disord. 130, 454-461) (37)
	Age 45-64 years	0.06833333	Derived from Jokela et al. (2011, J. Affect. Disord. 130, 454-461) (37)

Input	Stratification	Value	Notes
	Age 65 years and over	0.06833333	Derived from Jokela et al. (2011, J. Affect. Disord. 130, 454-461) (37)
Pre-COVID-19 self-harm hospitalisation rate ratio psychological distress		10.00004	Derived from Chamberlain et al. (2009, Crisis 30, 39–42) (38)
**Intervention: Employment programs**			
Employment programs starting year		2020.33000000	Equivalent to end April 2020
Years to implement employment programs		0.166666667	Equivalent to two months
Employment programs duration		1	Equivalent to 1 year.
Effect of employment programs on employed to unemployed rate		0.56	Derived from Business Indicators, Business Impacts of COVID-19 (ABS survey, April 2020) (39)
Effect of employment programs on employment initiation rate		1	Equivalent to no effect of employment programs on employment initiation
**Intervention: Better Access**			
Services per patient increase starting year		2020.75	Equivalent to October 2020 as additional COVID-19 MBC mental health support commenced then.
Years to implement services per patient increase		0.166666667	Equivalent to two months
Services per patient increase duration		2.25	Equivalent to 2 years 3 months
Proportion of specialised services provided by psychiatrists	Major cities areas	0.376508398	Derived from data on Medicare-subsidised mental health services published by the Australian Institute of Health and Welfare (available at: https://www.aihw.gov.au/reports-data/health-welfare-services/mental-health-services/data) (11)
	Regional and remote areas	0.279133644	Derived from data on Medicare-subsidised mental health services published by the Australian Institute of Health and Welfare (available at: https://www.aihw.gov.au/reports-data/health-welfare-services/mental-health-services/data) (11)
Pre-intervention services per patient	Major cities areas	5.13103948	Derived from data on Medicare-subsidised mental health services published by the Australian Institute of Health and Welfare (available at: https://www.aihw.gov.au/reports-data/health-welfare-services/mental-health-services/data) (11)
	Regional and remote areas	4.073157206	Derived from data on Medicare-subsidised mental health services published by the Australian Institute of Health and Welfare (available at: https://www.aihw.gov.au/reports-data/health-welfare-services/mental-health-services/data) (11)
Additional psychiatrist and allied services per patient		4	People will attend an additional 4 consultations per year when the cap on the number of consultations per patient is increased.
Better Access services per week		1	Patients attend 1 consultation per week
**Intervention: Specialised mental health service capacity growth**			

Input	Stratification	Value	Notes
Specialised mental health service capacity increase starting year		2022	Equivalent to January 2022
Post-intervention specialised mental health service capacity increase per year multiplier		2	Increase annual capacity annual growth rate by 100%

CMHC	Community Mental Health Care
COVID-19	Coronavirus disease 2019
ED	Emergency Department
GP	General practitioner
Post-sec	Post-secondary
Psychopathol.	Psychological
Underempl.	Underemployed
Youth	People aged 15-24 years
Direct Access x%	Refers to allowing x% of help-seeking people to use the Direct Access program
n x Services growth rate	Corresponds to multiplying by n the annual growth rate of specialised mental health care consultations

Figure 11. Adverse mental health outcome estimates derived from the model and corresponding historical data from HealthStats NSW (8-9)and AIHW (10)

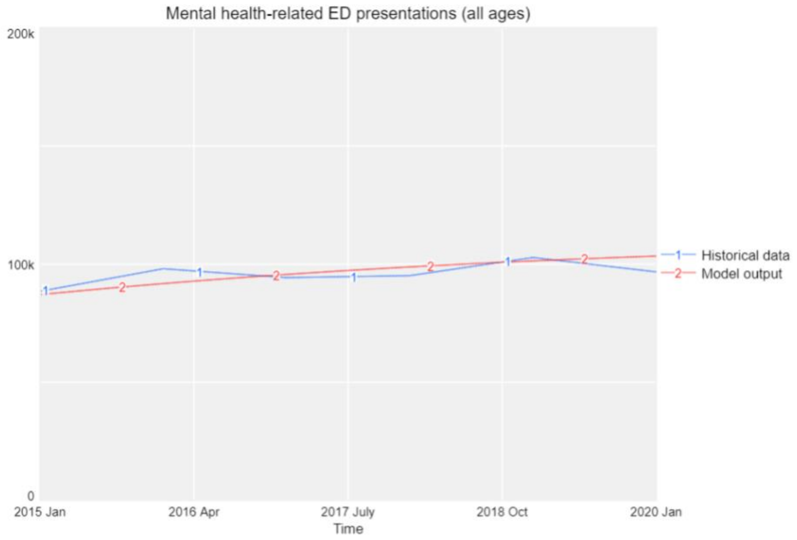

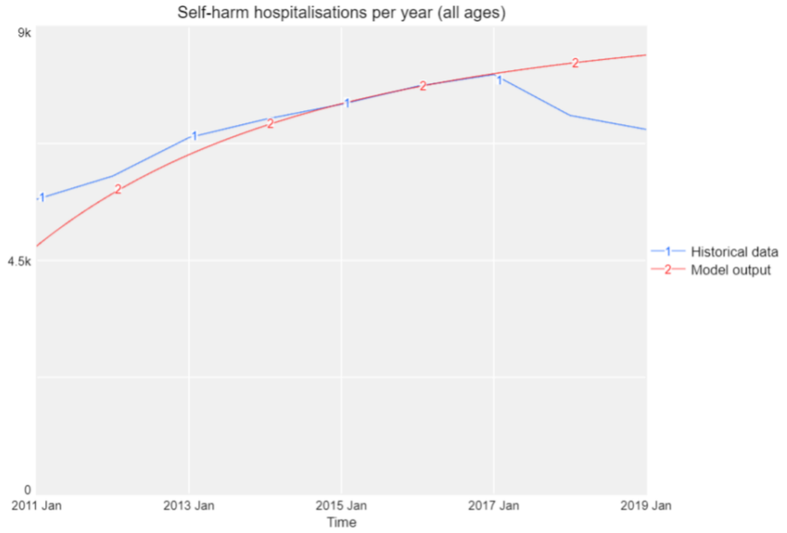

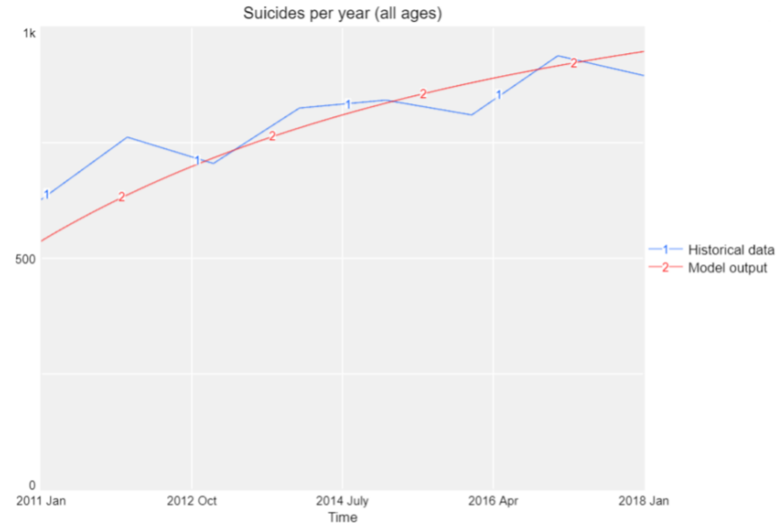

Table 2. Interventions and default parameter values. Parameter values could be modified via an interactive dashboard to assess the impact of different parameter values on simulated outputs

Intervention	Description
**Direct Access program**	Program designed to enable a proportion of help-seeking people to have access to subsidized sessions with psychologists or allied workers without requiring a general practitioner referral and mental health plan. Parameters that can be modified are:<br>*Starting year* — the year in which the Direct Access program commences (the default is 2022, or January 2022. When enabled in combination with specialised mental health service capacity growth, the starting year was changed to 2024, or January 2024).<br>*Implementation time (years)* — the time required for the Direct Access program to be fully implemented (the default is 2 years).<br>*Program duration (years)* — the duration of the Direct Access program (the default is 1000 years, ensuring the Direct Access program is enabled until the end of the simulation).<br>*Maximum use of Direct Access* — maximum proportion of help-seeking people having access to subsidized sessions with psychologists or allied workers without requiring a general practitioner referral and mental health plan. For this intervention, we varied to value from 0.1 to 0.5, corresponding to 10% to 50% of help-seeking people.
**Specialised mental health service capacity growth**	Multiplies the annual rate of increase in the total number of psychologists, psychiatrists and allied services that can be provided per week. The default value (1) corresponds to the business as usual case, in which service capacity continues to increase at the current rate, estimated using Medicare Benefits Schedule (MBS) data for 2014-2019 assuming services were operating at (near) maximum capacity over this period. For this intervention, we multiplied the annual rate of increase by successively 2, 3 and 5.<br>This multiplicative increase in service capacity growth rates commences in January 2022 and remains in place until the end of the simulation.
**Increase in demand for mental-health services**	Increases (by 5% to 10%) the per capita rates at which people perceive a need for mental health services and seek help from a general practitioner, online services, and from psychologists and allied professionals if Direct Access is enabled.<br>Default duration: until end of simulation.
**Employment programs**	As employment programs were implemented by the Australian government in response to the COVID-19 pandemic, this intervention was enabled for the COVID-19 baseline scenario model calibration.<br>These employment programs are designed to stem rapidly increasing unemployment due to the COVID-19 pandemic (e.g., the JobKeeper Payment). This intervention reduces the increase in the per capita job loss rate resulting directly from the

Intervention	Description
	pandemic. The per capita rate of employment initiation can also be increased (or decreased); however, the default settings assume that employment programs have no direct effect on employment initiation. Parameters that can be modified are:<br>*Starting year* — the year in which employment programs commence (the default is 2020.33 or end April 2020).<br>*Implementation time (years)* — the time required for employment programs to be fully implemented (the default is 0.167 years, or 2 months).<br>*Program duration (years)* — the duration of employment programs (the default is 1 year).<br>*Effect on job loss* — the multiplicative effect of employment programs on the increase in the job loss rate due to the COVID-19 pandemic. The default value (0.56) assumes that employment programs will reduce the increase in the per capita job loss rate by 44% (40).<br>*Effect on employment initiation* — the multiplicative effect of employment programs on the per capita employment initiation rate. The default value (1) assumes no effect of employment programs on employment initiation.
**Better Access**	As this program was implemented by the Australian government in response to the COVID-19 pandemic, this intervention was enabled for the COVID-19 baseline scenario model calibration.<br>Reform of the existing *Better Access to Psychiatrists, Psychologists and General Practitioners through the MBS* (Better Access) initiative to provide people with access to a greater number of specialised mental health care consultations per year. This intervention increases the flow of people with a perceived need for mental health care into psychiatrist and allied mental health services. Parameters that can be modified are:<br>*Starting year* — the year in which the reformed Better Access initiative commences (the default is 2020.75, or October 2020).<br>*Implementation time (years)* — the time after commencement required for the reformed Better Access initiative to be fully implemented (the default is 0.167 years, or 2 months).<br>*Program duration (years)* — the duration of the reformed Better Access initiative. The default is set to 2.25 years (2 years 3 months).<br>*Services per week* — the mean number of specialised mental health care services provided per patient per week. The default value (1) assumes that patients attend 1 consultation per week, so that a patient attending a total of 4 consultations (for example) is assumed to do so over a period of 4 weeks.<br>*Additional services per patient* — the mean number of additional specialized mental health care services provided per patient per year under the reformed Better Access scheme. The default value (4) assumes that patients will attend an additional 4 consultations per year when the cap on the number of consultations per patient is increased.

Table 3. Projected mental health-related emergency department presentations, self-harm hospitalisations and suicide deaths over the period September 2021 to September 2028 with and without COVID-19

	No COVID-19	COVID-19	Proportional change
Total NSW population			
Mental-health-related ED presentations	749568	738913	-1.42% (*)
Self-harm hospitalisations	62085	63198	1.79%
Suicide deaths	7455	7433	-0.29% (*)
NSW population aged 15-24 years			
Mental-health-related ED presentations	141128	141806	0.48%
Self-harm hospitalisations	17599	18453	4.85%
Suicide deaths	845	886	4.81%

(*) This decrease occurs because of lower population growth due to COVID-19 related border closures.

The population of NSW in 2028 is projected to be 3.9% lower than what would have been the case if the pandemic had not occurred because of the impact of COVID-19 on overseas and interstate migration (41). Despite this population decrease, Table 1 shows that self-harm hospitalisations are expected to increase by 1.79% for the general population (all ages), and by 4.85% in the 15-24 years age group relative to the no-COVID-19 scenario. These projections highlight that the COVID-19 disruption will have a more severe impact on the mental health and well-being of younger people in coming years.

Table 4. Simulated impact of allowing direct access to a proportion of Medicare-subsidised specialised mental health services, together with an increase in specialised mental health service capacity, on population mental health indicators, 1 September 2021 – 1 September 2028: impact of retaining extended Better Access until 31 December 2023

Scenarios	**Mental health-related ED presentations prevented**	**Youth mental health-related ED presentations prevented**	**Self-harm hospitalisations prevented**	**Youth self-harm hospitalisations prevented**	**Suicide deaths prevented**	**Youth suicide deaths prevented**
Baseline (with COVID-19)	-1191 (-0.16%)-	-77 (-0.05%)-	-42 (-0.07%)-	-3 (-0.02%)-	-5 (-0.07%)-	-0.13 (-0.02%)-
x2 Growth rate specialised mental health-related services	7535 (1.02%)	882 (0.62%)	340 (0.54%)	50 (0.27%)	45 (0.61%)	2.4 (0.27%)
Direct Access 10% + 2x Growth rate specialised mental health-related services	8558 (1.16%)	1147 (0.81%)	368 (0.58%)	62 (0.34%)	48 (0.64%)	3 (0.34%)
x3 Growth rate Specialised mental health-related services	11530 (1.56%)	1221 (0.86%)	512 (0.81%)	64 (0.34%)	70 (0.94%)	3 (0.4%)
Direct Access 20% + 3x Growth rate specialised mental health-related services	16502 (2.23%)	2169 (1.53%)	721 (1.14%)	120 (0.65%)	94 (1.3%)	5.8 (0.65%)
x5 Growth rate specialised mental health-related services	15637 (2.11%)	1576 (1.11%)	669 (1.06%)	77 (0.42%)	93 (1.2%)	3.7 (0.42%)
Direct Access 50% + x5 Services growth rate	27765 (3.75%)	3389 (2.39%)	1224 (1.94%)	187 (1.01%)	161 (2.16%)	9 (1%)

Youth refers to people aged 15-24 years. Better Access with 10 additional sessions from October 2020 till end December 2023. The increase in specialised mental health services starts in 2022. The Direct Access program starts in 2024. "Direct Access x%" refers to allowing x% of help-seeking people to use the Direct Access program. "n x Services growth rate" corresponds to multiplying by n the annual growth rate of specialised mental health care consultations.

REFERENCES

1. Australian Bureau of Statistics. National, state and territory population. June 2020. https://www.abs.gov.au/statistics/people/population/national-state-and-territory-population/jun-2020 (accessed April 2021).
2. Australian Bureau of Statistics. Labor force. July 2021. https://www.abs.gov.au/statistics/labour/employment-and-unemployment/labour-force-australia/jul-2021 (accessed September 2021).
3. Australian Bureau of Statistics. Labor Force, Detailed. July 2021. https://www.abs.gov.au/statistics/labour/employment-and-unemployment/labour-force-australia-detailed/jul-2021 (accessed September 2021)
4. Australian Bureau of Statistics. Education and work. May 2020. https://www.abs.gov.au/statistics/people/education/education-and-work-australia/may-2020 (accessed January 2022).
5. Australian Bureau of Statistics. Family characteristics and transitions. 2012-2013. https://www.abs.gov.au/statistics/people/people-and-communities/family-characteristics-and-transitions/latest-release (accessed April 2021).
6. Australian Bureau of Statistics. Household Impacts of COVID-19 Survey. June 2021. https://www.abs.gov.au/statistics/people/people-and-communities/household-impacts-covid-19-survey/jun-2021 (accessed July2021).
7. Centre for Epidemiology and Evidence. HealthStats NSW. Sydney: NSW Ministry of Health. https://www.healthstats.nsw.gov.au/#/r/102943 (accessed July 2021).
8. Centre for Epidemiology and Evidence. HealthStats NSW. Sydney: NSW Ministry of Health. Available at: https://www.healthstats.nsw.gov.au/#/r/107184 (accessed July 2021)
9. Centre for Epidemiology and Evidence. HealthStats NSW. Sydney: NSW Ministry of Health. Available at: https://www.healthstats.nsw.gov.au/#/r/107183 (accessed July 2021)
10. Australian Institute of Health and Welfare. Emergency department care tables. 2020. https://www.aihw.gov.au/reports-data/myhospitals/content/data-downloads (accessed on July 2021).
11. Australian Institute of Health and Welfare. Mental Health Services. 2020. Medicare-subsidised mental health-specific services, community mental health care services, same day mental health-related care, overnight admitted mental health-related care and private hospital-based ambulatory psychiatric services tables. https://www.aihw.gov.au/reports-data/health-welfare-services/mental-health-services/data (accessed July2021).
12. Australian Early Development Census. Data Explorer. 2018. https://www.aedc.gov.au/data/data-explorer (accessed October 2020).

13 Australian Bureau of Statistics. National, State and Territory Population. June 2020. https://www.abs.gov.au/statistics/people/population/national-state-and-territory-population/jun-2020 (viewed April 2021).

14 Australian Bureau of Statistics. 2012. Information paper: Use of the Kessler psychological distress scale in ABS health surveys, Australia, 2007-08. Cat. no. 4817.0.55.001. https://www.abs.gov.au/how-cite-abs-sources (viewed April 2021)

15 Dooley D, Prause J, Ham-Rowbottom KA. Underemployment and depression: longitudinal relationships. *J Health Soc Behav*. 2000;41(4):421-436.

16 Green MJ, Tzoumakis S, Laurens KR, et al. Early developmental risk for subsequent childhood mental disorders in an Australian population cohort. *Aust N Z J Psychiatry*. 2019;53(4):304-315.

17 Australian Bureau of Statistics. 2018. National Health Survey: first results, 2017-18. Cat. no. 4364.0.55.001. https://www.abs.gov.au/statistics/health/health-conditions-and-risks/national-health-survey-first-results/latest-release (veiwed April 2021)

18 Dean K, Green MJ, Laurens KR, et al. The impact of parental mental illness across the full diagnostic spectrum on externalising and internalising vulnerabilities in young offspring. *Psychol Med*. 2018;48(13):2257-2263.

19 Australian Bureau of Statistics. 2012-13. *Family characteristics and transitions*. https://www.abs.gov.au/statistics/people/people-and-communities/family-characteristics-and-transitions/latest-release. (viewed July 2021)

20 Australian Bureau of Statistics. 2019. *Education and Work, Australia*. https://www.abs.gov.au/statistics/people/education/education-and-work-australia/may-2019. (viewed July 2021)

21 Backlund E, Sorlie PD, Johnson NJ. A comparison of the relationships of education and income with mortality: the National Longitudinal Mortality Study. *Soc Sci Med*. 1999;49(10):1373-1384.

22 Lee S, Tsang A, Breslau J, et al. Mental disorders and termination of education in high-income and low- and middle-income countries: epidemiological study [published correction appears in Br J Psychiatry. 2011 Apr;198(4):327]. *Br J Psychiatry*. 2009;194(5):411-417.

23 Australian Bureau of Statistics. 2019. *Labour Force, Australia*. https://www.abs.gov.au/statistics/labour/employment-and-unemployment/labour-force-australia/dec-2019. (viewed April 2021)

24 Wilkins, RB. Personal and Job Characteristics Associated with Underemployment. *Aust. J. Labour Econ.* 2006. 9: 371-393.

25 Australian Bureau of Statistics. 2019, December. *Labour Force, Australia, Detailed*. https://www.abs.gov.au/statistics/labour/employment-and-unemployment/labour-force-australia-detailed/dec-2019. (viewed April 2021)

26 Frijters P, Johnston DW, Shields MA. The effect of mental health on employment: evidence from Australian panel data. *Health Econ*. 2014;23(9):1058-1071.

27 Australian Bureau of Statistics. 2020, May. *Education and Work, Australia*. https://www.abs.gov.au/statistics/people/education/education-and-work-australia/may-2020. (viewed April 2021)

28 Wilkins. The extent and consequences of underemployment in Australia. Melbourne Institute working paper no. 16/04. The University of Melbourne. 2004. https://melbourneinstitute.unimelb.edu.au/downloads/working-paper-series/wp2004n16.pdf (Viewed Jan 2021)

29 Wilkins. Personal and Job Characteristics Associated with Underemployment. *Aust J Labour Econ*. 2006. 9(4): 371-393

30 Christensen H, Griffiths KM, Jorm AF. Delivering interventions for depression by using the internet: randomised controlled trial. *BMJ*. 2004;328(7434):265.

31 Tyrer P, Morgan J, Van Horn E, et al. A randomised controlled study of close monitoring of vulnerable psychiatric patients. *Lancet*. 1995;345(8952):756-759.

32 Kessler RC, Zhao S, Blazer DG, Swartz M. Prevalence, correlates, and course of minor depression and major depression in the National Comorbidity Survey. *J Affect Disord*. 1997;45(1-2):19-30.

33 Cuijpers P, van Straten A, van Schaik A, Andersson G. Psychological treatment of depression in primary care: a meta-analysis. *Br J Gen Pract*. 2009;59(559):e51-e60.

34 Handley TE, Inder KJ, Kelly BJ, et al. You've got to have friends: the predictive value of social integration and support in suicidal ideation among rural communities. *Soc Psychiatry Psychiatr Epidemiol*. 2012;47(8):1281-1290.

35 Dooley D, Catalano R. Recent research on the psychological effects of unemployment. *J Soc Issues*. 1988 Jan;44(4):1-2.

36 Australian Bureau of Statistics. 2020, May. *Education and Work, Australia*. https://www.abs.gov.au/statistics/people/education/education-and-work-australia/may-2020. (viewed April 2021)

37 Jokela M, Singh-Manoux A, Shipley MJ, et al. Natural course of recurrent psychological distress in adulthood. *J Affect Disord*. 2011;130(3):454-461.

38 Chamberlain P, Goldney R, Delfabbro P, Gill T, Dal Grande L. Suicidal ideation. The clinical utility of the K10. *Crisis*. 2009;30(1):39-42.

39 Australian Bureau of Statistics. 2020, April. *Business Indicators, Business Impacts of COVID-19*. https://www.abs.gov.au/statistics/economy/business-indicators/business-conditions-and-sentiments/apr-2020. (viewed May 2021)

40. Australian Bureau of Statistics. Business Indicators, Business Impacts of COVID-19 (Reference Period: April 2020). 2020.

41. Population Statement 2020 state and territory population projections. 2020. https://population.gov.au/publications/statements/2020-population-statement (accessed May 2021).
